# Physicochemical convergence in antibody CDR3-VH repertoires recognizing phosphorothioate-modified oligonucleotides backbone

**DOI:** 10.3389/fimmu.2026.1843118

**Published:** 2026-05-29

**Authors:** Riccardo Galasso, Francesco Coppolino, Alessia Berbiglia, Grete Francesca Privitera, Agata Famà, Giuseppe Valerio De Gaetano, Germana Lentini, Concetta Beninati

**Affiliations:** 1 Department of Human Pathology of the Adult and Developmental Age “G. Barresi”, University of Messina, Messina, Italy; 2 Scylla Biotech s.r.l., Messina, Italy; 3 Department of Clinical and Experimental Medicine, Bioinformatic Unit, University of Catania, Catania, Italy

**Keywords:** antisense oligonucleotides, complementarity-determining region, next generation sequencing, phosphorothioate modifications, physicochemical signatures, single-chain variable fragments

## Abstract

**Introduction:**

The development of recombinant antibody binders against phosphorothioate-modified antisense oligonucleotides (PS-ASOs) remains challenging due to the highly polyanionic character and structural flexibility of the phosphorothioate backbone. Here, we applied a next-generation sequencing (NGS)-guided phage display strategy to determine whether selection against PS-modified ASOs induces reproducible repertoire remodeling and the emergence of shared CDR3 physicochemical signatures associated with PS-ASO recognition.

**Methods:**

Two independent two-round phage display biopanning strategies against biotinylated PS-ASO targets were coupled to targeted amplicon sequencing of VH and VL FR3–CDR3–FR4 regions on the Illumina MiSeq platform. CDR3 clonotypes were reconstructed using a dedicated bioinformatic pipeline including quality filtering, read merging, in-frame translation, clonotype counting, CPM normalization, enrichment analysis, and physicochemical descriptor profiling. Representative enriched scFv clones were further evaluated by ELISA-based binding and competition assays and by fluorescence microscopy in ASO-treated cells.

**Results:**

Deep sequencing revealed a marked reduction in repertoire diversity from Round 1 to Round 2, associated with reproducible clonal dominance across independent selection strategies. These changes were already evident at early stages of selection. A shared enriched set of 113 CDR3-VH clonotypes was identified and displayed a defined physicochemical profile, including increased positive charge, recurrent aromatic residue patterns, constrained CDR3-VH length distribution, higher theoretical pI, and reduced hydrophobicity. Representative functional assays further supported the relevance of this signature: among the selected recombinant scFv clones, 12F2 showed preferential binding to both PS1-ASO and PS2-ASO, with reduced reactivity toward the phosphodiester-backbone oligonucleotide used as control. In ASO-treated cells, 12F2 produced a detectable intracellular signal after PS-ASO transfection, whereas PO-ASO-treated cells showed absent or nearly absent signal.

**Discussion:**

These results define an NGS-guided framework for identifying early-stage repertoire focusing and physicochemical signatures associated with recognition of modified nucleic acid backbones. The common property-level features suggest convergent binding solutions compatible with recognition of phosphorothioate-associated molecular features, supporting the rational prioritization of candidate binders against challenging polyanionic targets.

## 1 Introduction

Antisense oligonucleotides (ASOs) incorporating phos-phorothioate (PS) backbone modifications have emerged as a major class of therapeutic nucleic acids due to their enhanced nuclease resistance, improved pharmacokinetics, and broad clinical applicability (1–3). Despite their widespread use, the development of high-affinity and sequence-independent antibodies recognizing PS-ASOs remains technically challenging. The phosphorothioate backbone confers a highly polyanionic character and structural flexibility, complicating the generation of antibodies with predictable binding behavior. Antibodies targeting nucleic acids have historically been studied in the context of anti-DNA autoimmunity, where structural and functional analyses have revealed distinctive binding mechanisms (4, 5). In particular, positively charged residues—especially arginine—within the heavy-chain complementarity-determining region 3 (CDR3-VH) have been shown to play a central role in mediating electrostatic interactions with the negatively charged phosphate backbone of DNA (6). Structural studies further demonstrated that antibody–DNA recognition may involve aromatic stacking interactions within CDR3-VH loops (7). Although PS-ASOs differ chemically from native DNA, both share a highly polyanionic backbone architecture, suggesting that similar physicochemical principles may govern antibody recognition. Collectively, these observations support the concept that the recognition of nucleic acids by antibodies is often strongly influenced by physicochemical factors.

The development of recombinant antibody technologies, including single-chain variable fragments (scFvs), has enabled the targeted selection of antibody fragments using phage display platforms. Phage display–derived scFvs have previously been employed to generate anti-idiotypic antibody fragments capable of mimicking complex non-protein antigens, including carbohydrate epitopes, demonstrating the versatility of scFvs in recognizing structurally disparate targets (8–10). Recent work has demonstrated the feasibility of generating antibodies capable of recognizing modified oligonucleotides, including PS-modified ASOs (11). However, while these studies validated binding specificity and diagnostic applicability, the evolutionary dynamics and molecular convergence of antibody repertoires during selection remain largely unexplored. Next-generation sequencing (NGS) enables high-resolution analysis of antibody repertoires, allowing quantitative monitoring of clonal enrichment, diversity contraction, and repertoire remodeling during selection. NGS-based approaches have also been successfully integrated with phage display-derived libraries to enable high-throughput mapping of antibody–antigen interactions and rapid characterization of antigenic determinants recognized by monoclonal or polyclonal antibodies (12–15).

In this study, we applied an NGS-guided analytical framework to examine the early evolution of CDR3 repertoires during phage display selection against PS-ASOs. Rather than aiming at exhaustive lead-binder characterization, we focused on the transition between Round 1 and Round 2 to determine whether early selection already imposes reproducible enrichment, repertoire focusing, and physicochemical convergence across independent selection strategies. By integrating quantitative enrichment analysis, descriptor-based profiling, and targeted functional validation, we investigated whether selection against PS-modified oligonucleotide backbones favors sequence-diverse but physicochemically convergent antibody binding solutions.

## 2 Materials and methods

### 2.1 Phage display selection strategies against PS-ASOs

Selections were performed using a human semi-synthetic library of scFvs displayed on the surface of filamentous M13 phage as polypeptides fused to the phage protein pIII. The scFv format consists of an immunoglobulin variable heavy (VH) and variable light (VL) domain joined by a flexible hydrophilic peptide linker. The VH chain of the scFv phage display library consists of a constant germline V segment (DP47), to which completely randomized sequences of four, five, or six amino-acids were appended by polymerase chain reaction with degenerate primers (16). Using this approach, combinatorial diversity was introduced at the level of CDR3-VH, a region known to play a major role in antigen recognition. The VL domain was based on the DPL16 germline segment, with a partially degenerate CDR3-VL region of fixed six-amino-acid length. Both CDR3 regions are flanked by invariant framework regions, referred to as FR3 upstream and FR4 downstream. Semi-synthetic scFv phage display libraries have been widely used because of their structural stability and their capacity to support extensive diversification within the CDR3-VH region, thereby increasing library diversity and the number of potential antigen-binding solutions (9, 17, 18). Accordingly, CDR3-VH and CDR3-VL were analyzed separately.

Antisense oligonucleotides were synthesized by IDT (Integrated DNA Technologies, Inc.). A 20-nt oligonucleotide fully modified with phosphorothioate inter-nucleotide linkages, named PS1-ASO, was designed as the first target. A second 20-nt oligonucleotide, named PS2-ASO, was also fully modified with phosphorothioate linkages and had the same purine/pyrimidine content as PS1-ASO but a scrambled nucleotide sequence. Both PS-modified ASOs used in this study carried a 3′ biotin modification to allow capture by streptavidin-coated magnetic beads. Two independent selection strategies were implemented, referred to as Selection 1 (S1) and Selection 2 (S2), each comprising two consecutive rounds of biopanning, referred to as Round 1 (R1) and Round 2 (R2). In Selection 1, PS1-ASO was used in both rounds of biopanning. In Selection 2, PS2-ASO was used in Round 1 and PS1-ASO in Round 2. This experimental design was intended to favor the identification of enrichment patterns associated with shared PS-backbone-related features rather than strictly sequence-specific recognition. Before target-mediated biopanning, and in order to reduce non-specific binders against the selection platform, approximately 10¹¹ phages derived from the scFv phage display library were pre-incubated with 50 µL of Dynabeads M-280 Streptavidin (Thermo Fisher) in 500 µL of PBS (137 mM NaCl, 2.7 mM KCl, 10 mM Na_2_HPO_4_, 1.8 mM KH_2_PO_4_) supplemented with 0.4% bovine serum albumin (BSA) and 0.1% Tween-20 for 30 min under rotation. After incubation, bead-associated phages were removed using a magnetic rack, while the unbound fraction, depleted of platform-reactive phages, was recovered and used as the input phage library for target selection. This pre-clearing step was specifically introduced to deplete phages recognizing beads, streptavidin-, or matrix-associated determinants and thereby reduce background binding related to the selection platform. For each round of biopanning, 400 nM biotinylated PS-ASO was incubated with the pre-cleared input phage library or with Round 1-selected phages overnight at 4 °C under rotation. To capture target-bound phages, 100 µL of Dynabeads M-280 Streptavidin were blocked for 1 h at room temperature in PBS supplemented with 1% BSA and 0.1% Tween-20 and subsequently added to the phage–PS-ASO mixture for 1 h at room temperature under rotation. Reactive phages were captured through the biotin–streptavidin interaction. After capture, beads were washed to remove unbound and weakly associated phages. Bound phages were eluted using 1 mL of 100 mM triethylamine (TEA) for 10 min and immediately neutralized with 0.5 volumes of Tris-HCl, pH 7.4. Eluted phages were amplified by infecting exponentially growing *Escherichia coli* TG1 cells (OD_600nm_ 0.2–0.4) in 2×TY medium for 30 min at 37 °C. Approximately 10¹¹ amplified phages were used for the subsequent round of selection under the same experimental conditions. After each round, selected phage pools were recovered for downstream NGS analysis and functional evaluation. The selection strategy was designed to capture early enrichment dynamics and repertoire remodeling between R1 and R2, rather than to perform exhaustive lead optimization through multiple rounds of biopanning.

### 2.2 Amplicon design and NGS library preparation

Phage-Seq analysis was performed on CDR3-containing regions, obtained by individual amplification of FR3–CDR3–FR4 regions of both VH and VL chains. Throughout the manuscript, these regions are referred to as CDR3-VH and CDR3-VL. Phage DNA was extracted from phage-infected *Escherichia coli*TG1 cells from the unselected scFv-phage display library (LibNS) and from selected phage pools (Selection 1-Round 1, Selection 1-Round 2, Selection 2-Round 1 and Selection 2-Round 2) using the Plasmid Mini kit (Qiagen) according to the manufacturer’s instructions. Our NGS-library preparation approach involved a three-step PCR protocol with different primer pairs, designed to minimize amplification bias while preserving repertoire diversity. A schematic overview of the strategy used for generation of CDR3 amplicons and NGS-library preparation is provided in **Supplementary Figure 1**, while the list of primers used in this study is shown in **Table 1**. Each phage DNA sample was amplified in triplicate to cover sequence diversity within each phage pool, employing a limited number of PCR cycles to reduce stochastic amplification effects.

**Table 1:** Primers used for the preparation of amplicon-based NGS libraries.

Pool	ID	Primers name	Sequence (5’ - 3’)	Expected amplicon size (bp)
First PCR primers				
	P1	VHeavy_CDR3_FW	GCTATTAGTGGTAGTGGTGGTAG	323 ± 3
	P2	VHeavy_CDR3_RV	CTGTCTCCTTGGCATGTGAT	323 ± 3
	P3	VLight_CDR3_FW	GATCCCAGACCGATTCTCTG	205
	P4	VLight_CDR3_RV	GTCGTCGTCCTTGTAGTCATC	205
Second PCR primers				
VH primer pool	P5	Rd1SP_VHeavy_CDR3_FW	TCGTCGGCAGCGTCAGATGTGTATAAGAGACAGGCTATTAGTGGTAGTGGTGGTAG	390 ± 3
	P6	Rd2SP_VHeavy_CDR3_RV	GTGTCGTGGGCTCGGAGATGTGTATAAGAGACAGCTGTCTCCTTGGCATGTGAT	390 ± 3
	P7	Rd1SP_1N_VHeavy_CDR3_FW	TCGTCGGCAGCGTCAGATGTGTATAAGAGACAGNGCTATTAGTGGTAGTGGTGGTAG	391 ± 3
	P8	Rd2SP_1N_VHeavy_CDR3_RV	GTGTCGTGGGCTCGGAGATGTGTATAAGAGACAGNCTGTCTCCTTGGCATGTGAT	391 ± 3
	P9	Rd1SP_2N_VHeavy_CDR3_FW	TCGTCGGCAGCGTCAGATGTGTATAAGAGACAGNNGCTATTAGTGGTAGTGGTGGTAG	392 ± 3
	P10	Rd2SP_2N_VHeavy_CDR3_RV	GTGTCGTGGGCTCGGAGATGTGTATAAGAGACAGNNCTGTCTCCTTGGCATGTGAT	392 ± 3
	P11	Rd1SP_3N_VHeavy_CDR3_FW	TCGTCGGCAGCGTCAGATGTGTATAAGAGACAGNNNGCTATTAGTGGTAGTGGTGGTAG	393 ± 3
	P12	Rd2SP_3N_VHeavy_CDR3_RV	GTGTCGTGGGCTCGGAGATGTGTATAAGAGACAGNNNCTGTCTCCTTGGCATGTGAT	393 ± 3
	P13	Rd1SP_4N_VHeavy_CDR3_FW	TCGTCGGCAGCGTCAGATGTGTATAAGAGACAGNNNNGCTATTAGTGGTAGTGGTGGTAG	394 ± 3
	P14	Rd2SP_4N_VHeavy_CDR3_RV	GTGTCGTGGGCTCGGAGATGTGTATAAGAGACAGNNNNCTGTCTCCTTGGCATGTGAT	394 ± 3
	P15	Rd1SP_5N_VHeavy_CDR3_FW	TCGTCGGCAGCGTCAGATGTGTATAAGAGACAGNNNNNGCTATTAGTGGTAGTGGTGGTAG	395 ± 3
	P16	Rd2SP_5N_VHeavy_CDR3_RV	GTGTCGTGGGCTCGGAGATGTGTATAAGAGACAGNNNNNCTGTCTCCTTGGCATGTGAT	395 ± 3
VL primer pool	P17	Rd1SP_VLight_CDR3_FW	TCGTCGGCAGCGTCAGATGTGTATAAGAGACAGGATCCCAGACCGATTCTCTG	272
	P18	Rd2SP_VLight_CDR3_RV	GTGTCGTGGGCTCGGAGATGTGTATAAGAGACAGGTCGTCGTCCTTGTAGTCATC	272
	P19	Rd1SP_1N_VLight_CDR3_FW	TCGTCGGCAGCGTCAGATGTGTATAAGAGACAGNGATCCCAGACCGATTCTCTG	273
	P20	Rd2SP_1N_VLight_CDR3_RV	GTGTCGTGGGCTCGGAGATGTGTATAAGAGACAGNGTCGTCGTCCTTGTAGTCATC	273
	P21	Rd1SP_2N_VLight_CDR3_FW	TCGTCGGCAGCGTCAGATGTGTATAAGAGACAGNNGATCCCAGACCGATTCTCTG	274
	P22	Rd2SP_2N_VLight_CDR3_RV	GTGTCGTGGGCTCGGAGATGTGTATAAGAGACAGNNGTCGTCGTCCTTGTAGTCATC	274
	P23	Rd1SP_3N_VLight_CDR3_FW	TCGTCGGCAGCGTCAGATGTGTATAAGAGACAGNNNGATCCCAGACCGATTCTCTG	275
	P24	Rd2SP_3N_VLight_CDR3_RV	GTGTCGTGGGCTCGGAGATGTGTATAAGAGACAGNNNGTCGTCGTCCTTGTAGTCATC	275
	P25	Rd1SP_4N_VLight_CDR3_FW	TCGTCGGCAGCGTCAGATGTGTATAAGAGACAGNNNNGATCCCAGACCGATTCTCTG	276
	P26	Rd2SP_4N_VLight_CDR3_RV	GTGTCGTGGGCTCGGAGATGTGTATAAGAGACAGNNNNGTCGTCGTCCTTGTAGTCATC	276
	P27	Rd1SP_5N_VLight_CDR3_FW	TCGTCGGCAGCGTCAGATGTGTATAAGAGACAGNNNNNGATCCCAGACCGATTCTCTG	277
	P28	Rd2SP_5N_VLight_CDR3_RV	GTGTCGTGGGCTCGGAGATGTGTATAAGAGACAGNNNNNGTCGTCGTCCTTGTAGTCATC	277

The first PCR step was performed to amplify the target DNA containing CDR3 diversity; in this step gene-specific primers annealing within invariable FR3 and FR4 were used to amplify FR3–CDR3–FR4 regions of VH (~323 bp) and VL (205 bp) independently. For each PCR reaction mix, 3 ng of template DNA was amplified using 0.5 unit of Q5^®^ High-Fidelity DNA Polymerase (New England Biolabs) with 200 µM of dNTPs and 100 nM of primers P1 and P2 for CDR3-VH, or P3 and P4 for CDR3-VL. Thermal cycling profile consisted of 20 cycles at 98 °C, 55 °C and 72 °C for 15 sec each. Subsequently, amplicons from each technical replicate were purified using the PCR purification kit (Qiagen), quantified by dsDNA Quantification Assay (Qubit) and visualized on 1% agarose gel to evaluate PCR success.

The second PCR step was performed to add the consensus regions required for sequencing and introduce “heterogeneity spacers” to avoid optical errors due to excessive fluorescence during the reading of constant sequences. Two different primer pools were used for CDR3-VH and CDR3-VL (**Table 1**). Each pool consisted of 12 degenerate primers, including both forward and reverse primers which bind to the first PCR amplicons due to a partial complementarity with P1 to P4, and incorporate the Illumina sequencing consensus and the heterogeneity spacers (0–5 randomized nucleotides). These variable-length spacers increase base diversity during sequencing to reduce optical and base-calling artifacts associated with low-diversity amplicon libraries (19–21). For each PCR reaction mix, 2 ng of template DNA was amplified using 0.5 unit of Q5^®^ High-Fidelity DNA Polymerase with 200 µM of dNTPs and 100 nM of “VH primer pool” (from P5 to P16) or “VL primer pool” (from P17 to P28). Thermal cycling profile consisted of 15 cycles at 98 °C, 55 °C and 72 °C for 15 sec each. Subsequently, PCR amplicons derived from each technical replicate were purified using the PCR purification kit (Qiagen), quantified by dsDNA Quantification Assay (Qubit) and visualized on 1% agarose gel to evaluate PCR success. Technical replicates derived from each starting phage DNA were pooled at equimolar concentrations for the third PCR step.

The third PCR step was performed to multiplex individual samples on the same sequencing run, using the MiSeq Reagent Kit v3 (Illumina). For each PCR reaction mix, 9 ng of template DNA was amplified using EPM Master Mix and 5 µL of dual-index specific primers (Illumina DNA/RNA UD Indexes Set A). Primers bound to the second PCR amplicons due to a partial complementarity with the previously used primer pools and introduces a 10-nt dual-index barcode and the appropriate Illumina adapter allowing amplicon-libraries to bind to the flow cell. Thermal cycling profile consisted of 12 cycles at 98 °C, 62 °C and 68 °C for 45 sec, 30 sec and 2 min respectively. Amplified libraries were purified using double-sided magnetic bead purification (Illumina) to remove short fragments and primer dimers. Indexed amplicon-libraries were quantified by dsDNA Quantification Assay (Qubit) and visualized on 1% agarose gel.

To load the flow cell, amplicon-libraries were pooled equimolarly and diluted at a final loading concentration of 10 pM. PhiX control library was spiked into the amplicon library pool at 40% to artificially increase genetic diversity. Amplicon-libraries were paired-end (2 × 300 bp) sequenced on a MiSeq flow cell using a v3 MiSeq sequencing kit. Image analysis, base calling, and data quality assessment were performed on the MiSeq instrument. Raw sequencing reads are publicly available (see Data Availability Statement section).

### 2.3 Bioinformatic processing and clonotype quantification

Raw paired-end reads generated on the Illumina MiSeq platform were subjected to quality assessment using FastQC (v0.74+galaxy1), and reports were aggregated using MultiQC (v1.27+galaxy3). Quality filtering was performed using Trimmomatic (v0.39+galaxy2) with a 4-nt sliding-window approach and a minimum Phred score threshold of 28. No additional minimum read-length threshold was applied beyond amplicon-size filtering. Reads passing quality filtering with a minimum length after of 50 bp were merged using PEAR (v0.9.6.4) with a minimum overlap of 10 bp and a statistical significance threshold (*p* < 0.05). Only successfully merged reads within the expected amplicon size range were retained. Sequences containing premature stop codons, frameshifts, unexpected lengths, or lacking the expected flanking motifs were excluded from downstream clonotype analysis. CDR3 regions were identified at the amino-acid level based on invariant flanking motifs characteristic of the antibody variable regions. Using the R package stringr (v1.6.0), only sequences within the expected FR3–CDR3–FR4 length range were retained.

First of all, fasta nucleotide merged sequences where selected retaining only upstream FR3-related sequences “AGTGGTGGTAGCACA” for VH and “TTCTCTGGCTCCAGCT” for VL. After that, counts were translated in-frame according to the expected scFv reading frame using Transeq frame 1 (EMBOSS:6.6.0.0) (22). Secondly, amino-acid sequences where selected and CDR3-VH region was defined as the amino-acid sequence located between the FR3-VH motif VYYCAK and the FR4-VH motif FDYWGQ with a length of less than 19 amino-acids, whereas CDR3-VL region was defined as the amino-acid sequence located between the FR3-VL motif YYCN and the FR4-VL motif VVFG with a length of less than 16 amino-acids. Clonotypes were defined as unique CDR3 amino-acid sequences and analyzed independently for CDR3-VH and CDR3-VL datasets. Within each sample, identical CDR3 sequences were collapsed and counted to generate clonotype-level abundance tables. *Per*-sample raw reads, trimmed reads, merged reads, nucleotide sequences number after cut, amino-acidic number sequences and final collapsed clonotype counts are reported in **Supplementary Table 1**.

Clonotypes with cumulative counts (Round 1 + Round 2) < 5 were excluded to minimize stochastic noise arising from extremely low-frequency variants. This cutoff was chosen as a conservative filtering step to reduce the contribution of sporadic low-count sequences while preserving sufficient repertoire diversity for downstream analyses. To evaluate the robustness of this choice, sensitivity analyses were performed using alternative cumulative count thresholds (R1+R2 ≥ 2, ≥ 5, ≥ 10, and ≥ 20), as reported in **Supplementary Tables 2**, **S3**. Sensitivity analyses showed that the shared enriched CDR3-VH clonotype set was stable across thresholds of R1+R2 ≥ 2, ≥ 5, and ≥ 10, consistently yielding 113 clonotypes, whereas only the most stringent threshold tested (R1+R2 ≥ 20) reduced this set to 73 clonotypes. This supported the use of R1+R2 ≥ 5 as a conservative but non-over restrictive cutoff, able to reduce low-count stochastic noise while preserving the shared enriched clonotype set used for downstream analyses.

For downstream analyses, clonotype abundances were normalized as counts per million reads (CPM) to account for differences in sequencing depth between samples. Relative enrichment between R2 and R1 was calculated as log_2_ ((CPM_R2 + 1)/(CPM_R1 + 1)), where a pseudocount of 1 was introduced to avoid division by zero. Enrichment relative to the non-selected library (LibNS), where reported, was calculated using the same CPM-based normalization approach. To identify clonotypes reproducibly enriched across independent selection strategies, a shared enrichment score (E_shared) was defined as the minimum log_2_ enrichment observed for the same clonotype across Selection 1 and Selection 2 datasets. This minimum-based definition was intentionally conservative, as it retains clonotypes showing reproducible enrichment across both independent selection strategies rather than strong enrichment in only one condition. Sensitivity analyses comparing E_shared thresholds were performed to assess robustness (**Supplementary Table 4**). The E_shared > 4 threshold was selected as an intermediate stringency criterion, balancing reproducible enrichment with sufficient clonotype representation for descriptor-based comparisons. At this threshold, the shared enriched CDR3-VH set retained 113 clonotypes showing large effect-size differences and strong significativity for each physicochemical desctriptor, as reported in **Supplementary Table 4**. Clonotypes meeting predefined enrichment thresholds were retained for physicochemical profiling.

### 2.4 Statistical and computational analysis

All bioinformatic and statistical analyses were performed in the R environment (v4.5.2). Graphical visualizations were generated using ggplot2 (v4.0.0). For enrichment landscapes, raw counts were normalized as counts per million reads (CPM), calculated as the number of reads assigned to each clonotype divided by the total number of reads in the corresponding sample and multiplied by 10^6^.

Comparative enrichment between R2 and R1 was calculated as the log_2_ ratio between normalized counts in the two rounds, using the expression log_2_ ((CPM_R2 + 1)/(CPM_R1 + 1)), where a pseudocount of 1 was added to avoid division by zero. Rarefaction and diversity extrapolation analyses were implemented using custom R scripts based on Hurlbert’s analytical formulation and Chao1 richness estimators (23–26).

Rank–abundance analysis was generated by ranking clonotypes according to relative abundance within each dataset and plotting proportional frequencies against rank on log–log scales. Abundance summaries and proportional frequencies were computed using the dplyr package (v1.1.4). Regression slopes were used descriptively to compare repertoire focusing and clonal dominance between rounds.

Physicochemical descriptors of enriched CDR3 sequences were calculated using the Peptides R package (v2.4.6). For each CDR3-VH amino-acid sequence, the proportion of specific residue classes was computed by dividing the number of residues belonging to each category by the total CDR3-VH length. Residue classes included positively charged residues (K, R, H), negatively charged residues (D, E), aromatic residues (W, Y, F), and polar uncharged residues (S, T, N, Q, C). Theoretical isoelectric point (pI) and mean hydrophobicity were calculated using standard implementations within the Peptides package, with hydrophobicity values derived from the Kyte–Doolittle scale (27, 28). Descriptor values were analyzed on an unweighted clonotype basis, whereby each unique sequence contributed once to the analysis independently of its abundance, to evaluate intrinsic sequence properties. Density distributions were generated using Kernel density estimation applied to continuous descriptor values. Formal statistical comparisons between enriched clonotypes and the R1 reference repertoire were performed using the Mann–Whitney U test, as descriptor distributions were not assumed to be normally distributed. To account for multiple testing across descriptors, *p*-values were adjusted using the Benjamini–Hochberg false discovery rate (FDR) procedure. Rank-biserial correlation was calculated as an effect size measure for each descriptor comparison. All statistical comparisons were performed using effect size (v1.0.2). To assess primary sequence relatedness among enriched clonotypes, pairwise Levenshtein distances were calculated for the enriched CDR3-VH sequences. Where analyses focused on the randomized CDR3-VH core, invariant flanking residues were excluded before distance calculation. The resulting distance matrix was used to evaluate whether enriched clonotypes collapsed into a dominant sequence family or remained heterogeneous at the primary sequence level (**Supplementary Figure 5**). For positional analyses and sequence-logo visualization, CDR3-VH sequences were grouped according to randomized core length (4, 5, or 6 amino-acids) after removal of invariant flanking residues. Residue-class distributions and sequence logos were then generated separately for each length class to avoid alignment artifacts introduced by variable core length (**Supplementary Figure 4**). A parallel analysis was performed for CDR3-VL repertoires to evaluate whether light-chain CDR3 regions displayed comparable physicochemical shifts (**Supplementary Figure 6**).

Processed *per*-selection clonotype abundance tables and analysis scripts are available in the associated GitHub public data repository (see Data Availability Statement section).

### 2.5 ELISA-based functional assays

Functional validation of selected phage pools and recombinant scFv clones was performed by ELISA. Oligonucleotide called PO-ASO is a control oligonucleotide sharing the same nucleotide sequence of PS1-ASO, but presenting a natural phosphodiester backbone (PO). For phage ELISA, Phage pools were tested against immobilized biotinylated PS1-ASO, PS2-ASO, PO-ASO, and BSA-coated control wells to evaluate enrichment of PS-ASO-reactive binders during selection. Bound phages were first recognized by an anti-M13 primary antibody (Abcam, cat. A9225), followed by detection with an HRP-conjugated anti-mouse IgG secondary antibody (Bio-Rad, cat. 1721011). Signal development was achieved using TMB substrate (Bio-Rad). After TMB development, the reaction was stopped with 1 volume of 0.2 N H_2_SO_4_ and absorbance was measured at 450nm.

Representative scFv clones isolated from Round 2-enriched pools of Selection 2 were expressed in *Escherichia coli* HB2151 as soluble 6*His-FLAG-tagged recombinant fragments. Cultures were grown in 2×YT medium supplemented with ampicillin and induced with 1 mM IPTG at 25 °C overnight. Recombinant scFvs were recovered from bacterial lysates after lysozyme treatment, sonication, centrifugation, filtration, and purification using the Affi-Gel Protein A MAPS II Kit (Bio-Rad). Protein purity was assessed by SDS-PAGE and Coomassie staining, and concentrations were determined by Bradford assay.

For recombinant scFv capture ELISA, 96-well plates were coated overnight at 4 °C with anti-FLAG antibody (antibodies.com, cat. A58753) at 2 µg/mL in carbonate-bicarbonate buffer, pH 9.6. After blocking with 5% non-fat dry milk, purified scFvs were added at 5 µg/mL and incubated overnight at 4 °C. Biotinylated PS1-ASO, PS2-ASO, PO-ASO, or control molecules were then added at 0.05 µM for 2 h at room temperature, and detected using HRP-conjugated streptavidin (Bio-Rad, cat. STAR5B). After TMB development, the reaction was stopped with 1 volume of 0.2 N H_2_SO_4_ and absorbance was measured at 450nm.

Dose-dependent binding was evaluated using a direct ELISA format in which biotinylated PS-ASO or PO-ASO targets were immobilized on streptavidin-coated plates and incubated with increasing concentrations of recombinant scFv. Bound scFvs were detected using anti-6*His-HRP antibodies (Proteintech, cat. HRP-66005), followed by enzymatic development and absorbance reading at 450 nm. Apparent EC_50_ values were estimated from ELISA binding curves by nonlinear regression and defined as the concentration of scFv producing 50% of the maximal specific binding signal, calculated between the baseline/background signal and the upper plateau of the dose–response curve.

Competitive ELISA assays were performed under the scFv capture ELISA binding conditions, using a fixed concentration of PS1-ASO (0.01 µM), in the presence of increasing concentrations of soluble competitors. Competitors included sonicated Salmon Sperm DNA (Cytiva) as a polyanionic competitor, non-biotinylated PS-ASO, non-biotinylated PO-ASO, and BSA. Inhibition was calculated as signal reduction relative to wells incubated with PS1-ASO (0.01 µM) in the absence of competitor and expressed as residual binding or percentage inhibition.

### 2.6 Fluorescence microscopy in ASO-treated cells

Vero cells were seeded on μ-Slides VI 0.1 (Ibidi) and cultured under standard conditions before transfection with 2.5 µg/mL of PS-ASO or PO-ASO using DOTAP (Roche) as transfection reagent. Cells were analyzed at 6 h after transfection without medium replacement. Following transfection, cells were washed three times with DPBS and fixed with 4% paraformaldehyde for 15 minutes. Subsequently, cells were washed three additional times and permeabilized with 0.1% Triton X-100 for 10 minutes. Blocking was carried out for 1 hour at room temperature using a solution containing MaxBlock (Active Motif), 5% bovine serum albumin (BSA), FcR Block Reagent (Miltenyi Biotec) and goat serum. After blocking, slides were washed three times and incubated overnight at 4 °C under gentle agitation with purified recombinant scFv at 1 µg/mL diluted in TBS-T (Tris-Buffered Saline, Tris base 0.02M, NaCl 0.15M, supplemented with 0.1% Tween-20) containing 2% BSA. The following day, cells were incubated with an anti-6*His monoclonal antibody (Proteintech, cat. 10001-0-AP) for 1 hour. Bound His-tagged scFvs were detected using a goat anti-rabbit IgG Alexa Fluor 488-conjugated antibody (Thermo Fisher, cod. A-11008), and nuclei were counterstained with DAPI. Finally, ProLong reagent (Thermo Fisher), diluted 1:2 in Dulbecco’s PBS, was added to preserve fluorescence. Images were acquired using a Zeiss Axio Observer Z1 fluorescence microscope equipped with a 100x objective and ZEN imaging software. Exposure time and gain were kept constant across all samples.

## 3 Results

### 3.1 Experimental design and NGS workflow enable quantitative comparison of independent selections against PS-ASOs

**Figure 1:**
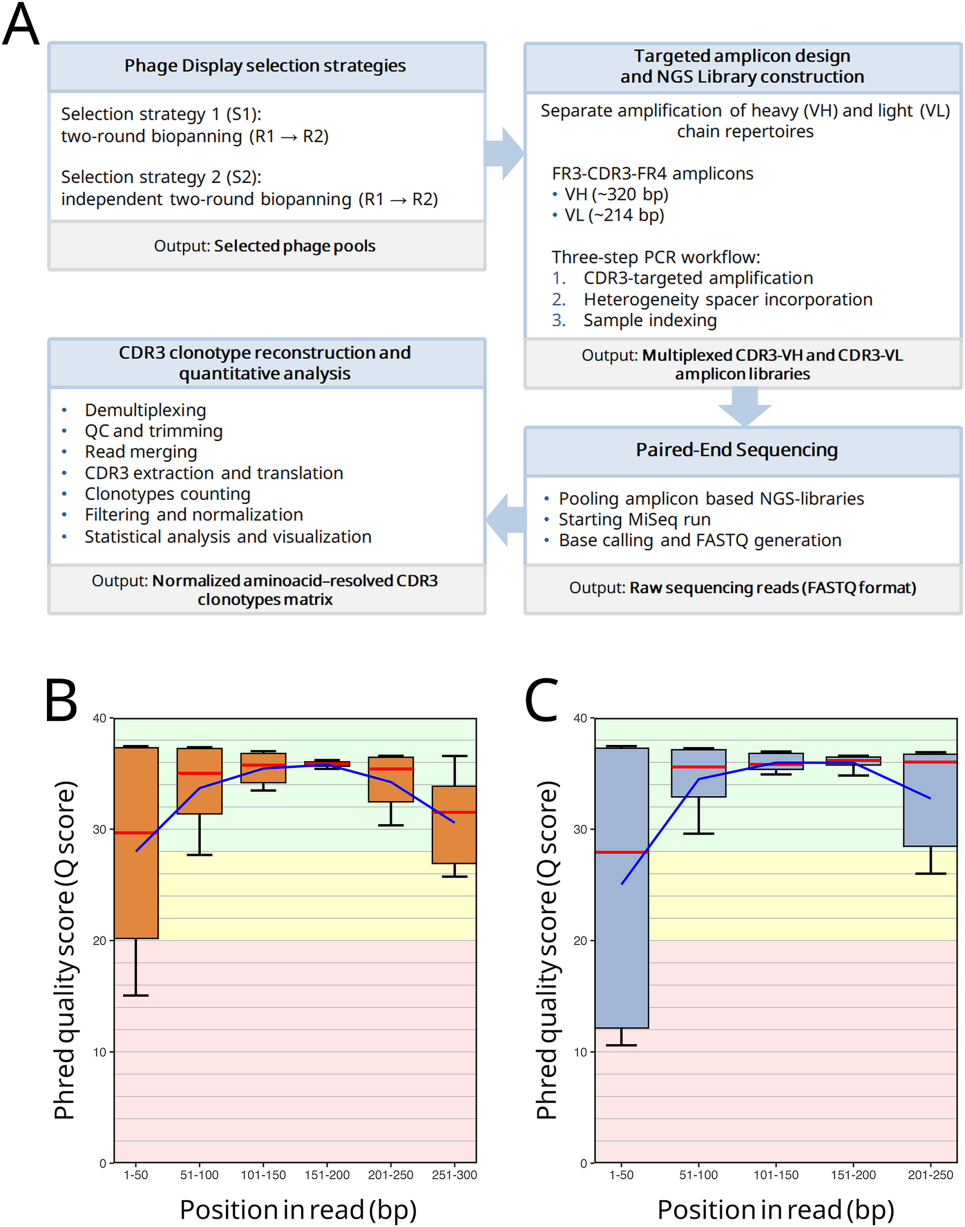
Experimental design and sequencing quality assessment. **(A)** Overview of the experimental workflow combining phage display selection and targeted NGS analysis of scFv repertoires. Two independent selection strategies (S1 and S2), each comprising two rounds of biopanning (R1 and R2), were performed. Selected phage pools were subjected to targeted amplification of the FR3–CDR3–FR4 regions of VH and VL domains, followed by next-generation sequencing and clonotype reconstruction. **(B, C)** *Per*-base Phred quality score distributions across read positions for aggregated CDR3-VH **(B)** and CDR3-VL **(C)** datasets. Box plots represent interquartile ranges, with medians indicated. Shaded regions denote Phred quality thresholds. Data represent combined sequencing reads from the libraries corresponding to the different selection rounds and strategies. Comparable quality profiles across read positions confirm high sequencing accuracy and technical consistency. Individual sample-level quality metrics are shown in **Supplementary Figure 2**.

We established an integrated experimental workflow combining scFv phage display selection against phosphorothioate-modified antisense oligonucleotides with targeted next-generation sequencing to quantitatively resolve scFv repertoire dynamics across independent selection strategies (**Figure 1A**). Two parallel selection strategies (S1 and S2) were performed, each comprising two consecutive rounds of biopanning (R1 and R2). In S1, the same phosphorothioate-modified oligonucleotide (PS1-ASO) was used in both rounds. In S2, two PS-modified oligonucleotides differing in nucleotide sequence but sharing the same phosphorothioate backbone chemistry were used (PS1-ASO and PS2-ASO). This experimental design enabled evaluation of whether enrichment patterns were primarily driven by recognition of the PS backbone rather than by sequence-specific interactions.

Importantly, the analysis was intentionally focused on the transition between Round 1 and Round 2, as this stage enables the capture of early selection-driven repertoire remodeling while preserving sufficient diversity for quantitative comparison. Selected phage pools from each round were subsequently processed for sequencing and downstream repertoire analysis. For chain-specific characterization of antibody repertoires, the FR3–CDR3–FR4 regions of the heavy (VH) and light (VL) variable chains were analyzed independently. The resulting amplicons contained CDR3 regions flanked by invariable framework sequences, enabling accurate identification and extraction of CDR3 amino-acid sequences for downstream analysis (**Figure 1A**). Sequencing yielded approximately 1.5 million paired-end reads per sample with consistently high sequencing quality. More than 85% of bases achieved Phred scores ≥ Q30. *Per*-base quality profiles averaged across all sequencing libraries showed highly consistent quality distributions along the read length for both VH and VL datasets (**Figures 1B, C**), supporting the absence of major technical bias prior to repertoire analysis. *Per*-sample quality profiles are shown in **Supplementary Figure 2**. To mitigate the low-diversity background typical of amplicon libraries, heterogeneity spacers were incorporated into the amplicon design. For this purpose, degenerate nucleotides (N, any base) were introduced immediately downstream of the sequencing primer-binding sites at both the 5′ and 3′ ends of the amplicons. Up to five variable bases were included to increase sequence diversity during sequencing, thereby improving cluster detection and base-calling accuracy on the Illumina platform. Following quality filtering, read merging, in-frame translation and CDR3 extraction, clonotypes were defined as unique CDR3 amino-acid sequences and counted independently for VH and VL datasets. Downstream analyses were then performed separately for VH and VL repertoires to enable comparative assessment of their contribution to selection-driven convergence. The consistent experimental and computational pipeline applied across all samples enabled robust quantitative comparison of clonal diversity, expansion dynamics, and enrichment patterns between independent selection strategies.

### 3.2 Rarefaction analysis reveals repertoire complexity and early contraction dynamics during selection against PS-ASOs

**Figure 2:**
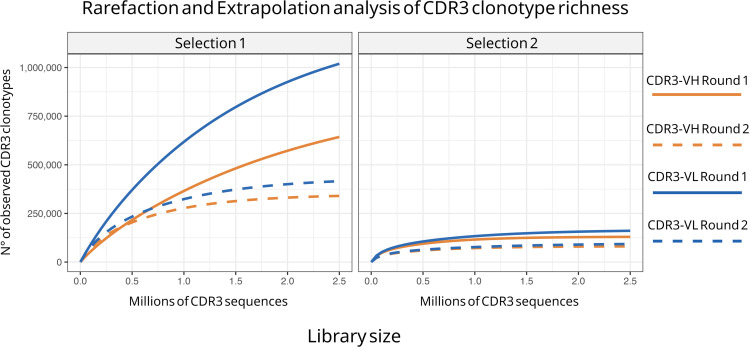
Rarefaction and extrapolation analysis of CDR3-VH and CDR3-VL repertoires across independent PS-ASO selection strategies. Rarefaction/extrapolation curves depict the cumulative number of unique CDR3 clonotypes as a function of sequencing depth. Left panel: Selection 1. Right panel: Selection 2. Solid lines represent Round 1 repertoires, whereas dashed lines represent Round 2 repertoires. Orange curves correspond to CDR3-VH datasets and blue curves to CDR3-VL datasets. Rarefaction analysis was performed using analytical subsampling to enable direct comparison across samples with different sequencing depths. The steeper initial slopes observed in Round 1 reflect higher intrinsic repertoire diversity, whereas reduced slopes and earlier plateauing in Round 2 indicate selection-driven repertoire contraction and clonal enrichment.

To quantitatively evaluate repertoire complexity and sequencing depth across independent selection strategies, rarefaction analysis was performed on unique CDR3 amino-acid clonotypes prior to abundance-based filtering. Rarefaction curves for S1 and S2 are shown in **Figure 2**. In both strategies, R1 repertoires exhibited substantially higher clonotype richness compared to R2 for both VH and VL chains. This pattern is consistent with the progressive reduction of low frequency clonotypes during the early stages of selection. The increase observed in R1 curves reflects the high intrinsic diversity of early selected pools, characterized by a large population of low frequency clonotypes. In contrast, R2 repertoires displayed reduced curve-related slopes and an earlier shift toward a plateau, indicating a contraction of repertoire diversity associated with enrichment processes occurring between Round 1 and Round 2. This pattern was observed in both independent selection strategies, indicating reproducible repertoire remodeling under PS-ASO–mediated selective pressure. Notably, S1 displayed substantially higher clonotype richness than S2 for both VH and VL repertoires, indicating that the two independent selection strategies generated repertoires with different baseline diversity levels. Despite these differences in absolute diversity levels, the transition from R1 to R2 consistently resulted in reduced complexity in both VH and VL datasets. Importantly, rarefaction curves approached an asymptotic trend within the achieved sequencing depth, supporting the conclusion that most detectable clonotype diversity was captured and enabling reliable comparison between selection rounds.

Collectively, these data demonstrate that PS-ASO selection induces measurable repertoire contraction already at early stages of biopanning, while sequencing depth was sufficient to monitor clonal dynamics across selection and biopanning strategies.

### 3.3 Selection-driven enrichment landscape of CDR3-VH clonotypes

The scFv-phage display library used in this study derives from germline immunoglobulin segments engineered to introduce completely randomized CDR3-VH sequences (16). Because CDR3-VH represents the major source of randomized diversity in this library, and consistent with previous studies indicating that the physicochemical properties of CDR3-VH play a central role in nucleic acid recognition (6, 7), subsequent enrichment landscape and convergence analyses were primarily focused on CDR3-VH clonotypes.

**Figure 3:**
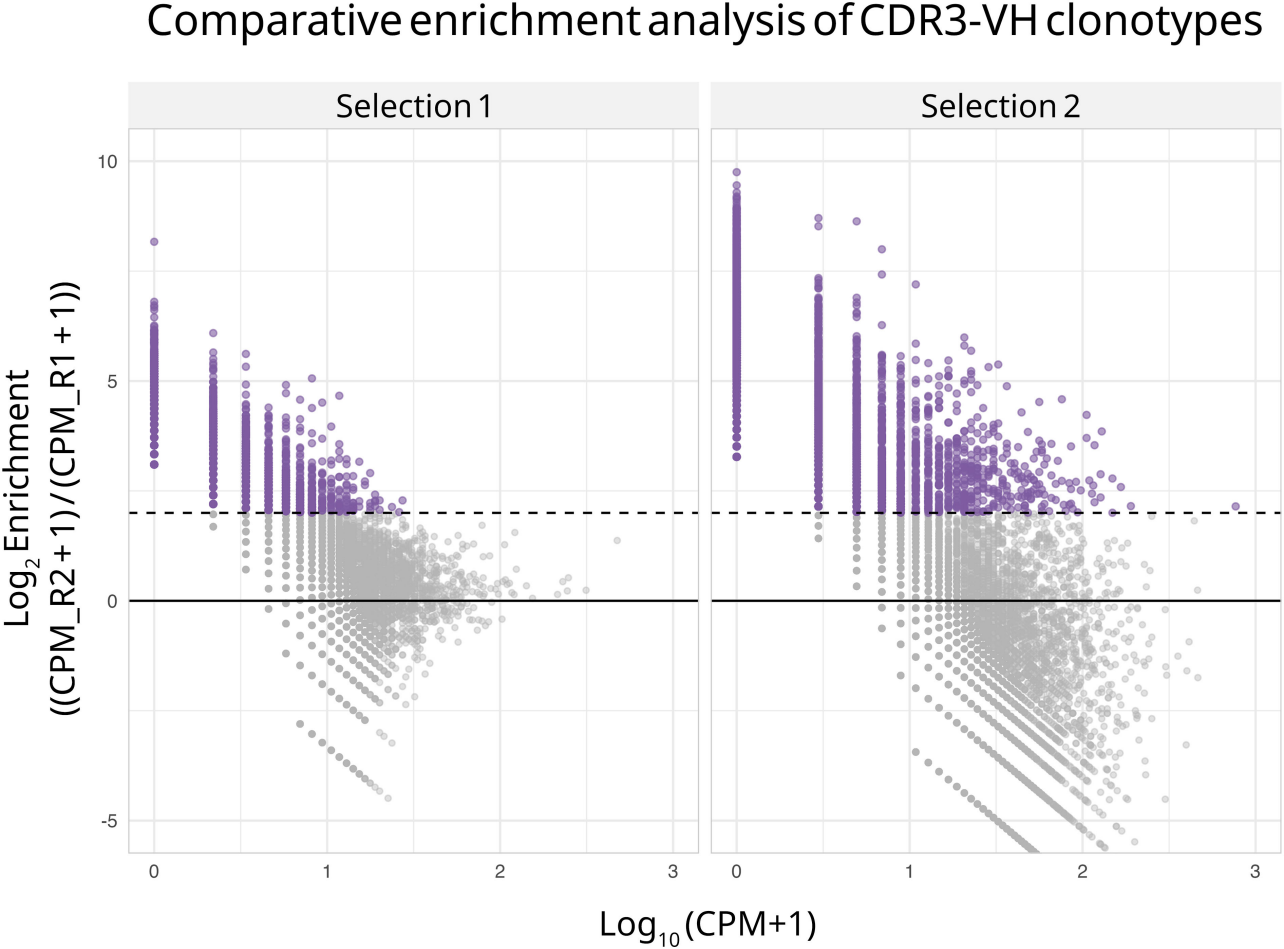
Selection-driven enrichment landscape of CDR3-VH clonotypes across independent PS-ASO selection strategies. Scatter plots depict enrichment dynamics of unique CDR3-VH clonotypes between Round 1 (R1) and Round 2 (R2). Left panel: Selection 1. Right panel: Selection 2. Each point represents a unique CDR3-VH amino-acid sequence. The x-axis shows the initial abundance in R1 expressed as log_10_(CPM + 1). The y-axis represents enrichment magnitude calculated as log_2_((CPM_R2 + 1)/(CPM_R1 + 1)), where a pseudocount of 1 was added to avoid division by zero. Only clonotypes with cumulative read counts (R1 + R2) ≥ 5 were included. Purple points denote clonotypes exceeding the enrichment threshold (log_2_ > 2), whereas gray points represent non-enriched or depleted sequences. The reproducible enrichment topology observed across independent strategies indicates consistent PS-ASO–driven selective pressure. CPM, counts per million.

To determine how phage display selection reshaped the antibody repertoire beyond the global contraction described in Section 3.2, we quantified changes in CDR3-VH clonotype abundance between R1 and R2 for each independent selection strategy. After quality filtering, sequences were translated and collapsed at the amino-acid level, to obtain unique CDR3-VH sequences corresponding to individual clonotypes. Clonotype abundance was normalized as counts per million reads (CPM), enabling quantitative comparison across rounds independently of sequencing depth. Relative enrichment between R2 and R1 was calculated as log_2_((CPM_R2 + 1)/(CPM_R1 + 1)), where a pseudocount of 1 was introduced to avoid division by zero. The enrichment landscapes for S1 and S2 are shown in **Figure 3**. Each dot represents a unique CDR3-VH amino-acid sequence plotted according to its initial abundance in R1 (x-axis, log_10_ (CPM + 1)) and its enrichment magnitude between rounds (y-axis, log_2_ enrichment). Across both selection strategies, the majority of clonotypes clustered around log_2_ enrichment ≈ 0, indicating limited abundance variation between rounds. However, a distinct subset of clonotypes exhibited strong positive enrichment, exceeding the pre-defined threshold of log_2_ > 2, thereby identifying a population of clonotypes preferentially expanded during selection. Conversely, a broad distribution of clonotypes displayed negative enrichment values, reflecting relative depletion during the selection process. This asymmetric enrichment pattern indicates that selection process selectively reshapes the repertoire by favoring a restricted subset of clonotypes rather than uniformly amplifying all sequences.

Importantly, the overall enrichment topology was broadly comparable across the two independent selection strategies, indicating reproducible enrichment trajectories under PS-ASO–driven selection conditions. Complementary enrichment distributions related to the non-selected library are shown in **Supplementary Figure 3** for both CDR3-VH and CDR3-VL repertoires across the two selection strategies and rounds. In these plots, enrichment values for R1 and R2 are displayed together for each dataset, allowing visualization of the progressive shift toward higher enrichment levels during selection.

Collectively, these results indicate that PS-ASO–driven selection induces structured and reproducible enrichment of specific CDR3-VH clonotypes, establishing a defined enrichment landscape that precedes the physicochemical convergence described in the following sections.

### 3.4 Rank–abundance analysis reveals progressive focusing of the CDR3-VH repertoire

**Figure 4:**
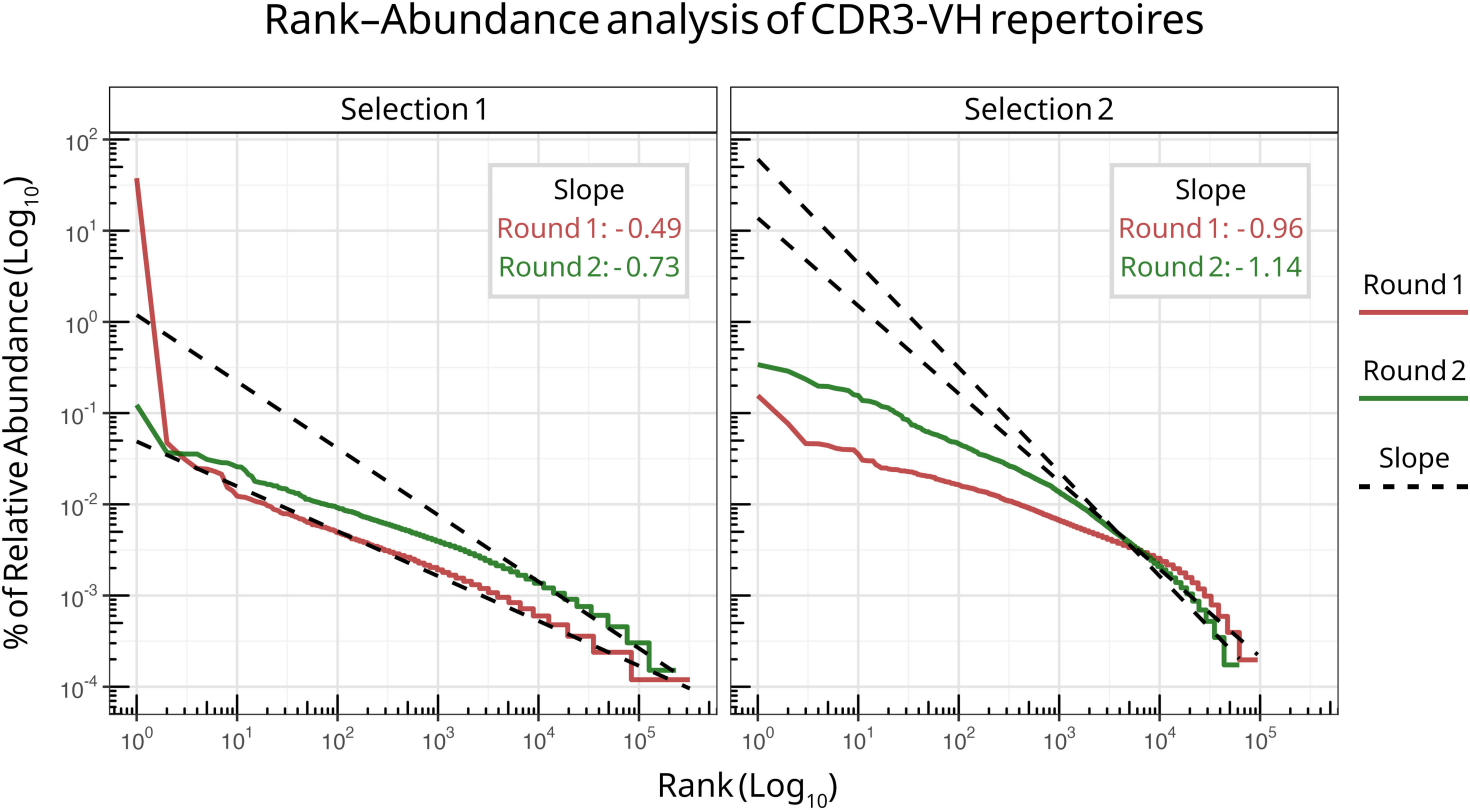
Rank–abundance analysis reveals progressive focusing of the CDR3-VH repertoire during PS-ASO selection. Rank–abundance (Whittaker) plots of CDR3-VH clonotypes for Selection 1 (left) and Selection 2 (right). Clonotypes were ranked according to sequence abundance, from the most abundant to the least abundant, and plotted against their relative abundance, expressed as percentage of total CDR3-VH sequences, using a log–log scale. Round 1 (R1) distributions are shown in red, and Round 2 (R2) distributions are shown in green. Dashed black lines represent linear regressions fitted to log-transformed distributions, with corresponding slope values indicated. The increased slope steepness observed in R2 reflects enhanced clonal dominance and reduced repertoire evenness following selection. The shift from R1 to R2 demonstrates a structured redistribution of clonotype frequencies, consistent with selective amplification of dominant CDR3-VH variants.

To evaluate how selection influenced clonal distribution beyond enrichment magnitude alone, we performed a rank-abundance analysis of the CDR3-VH repertoires (**Figure 4**). Repertoires from each round were plotted according to clonotype abundance rank, from most to least abundant, and relative abundance, expressed as percentage of total sequences, on a log–log scale. This representation allows a comparison of the repertoires from each round in terms of clonotype diversity, evenness, and richness.

In both selection strategies, R1 exhibited a comparatively flatter distribution, consistent with a broad and heterogeneous repertoire composed of many low frequency clonotypes. In contrast, R2 displayed a steeper slope, indicating increased dominance of a limited number of clonotypes and a reduction in repertoire evenness. Linear regression applied to the log-transformed distributions confirmed this shift quantitatively, supporting a redistribution of clonal abundance toward dominant clonotypes during the transition from Round 1 to Round 2. In S1, the slope shifted from −0.492 in R1 to −0.731 in R2, whereas in S2 the slope shifted from −0.962 to −1.143. This increase in slope steepness reflects progressive focusing of the repertoire, whereby a restricted subset of clonotypes becomes disproportionately represented following selection. Although the two independent selection strategies displayed different baseline slope values in R1, both showed a consistent shift toward steeper distributions after the second round of selection. Importantly, this focusing effect is already evident at the early stages of biopanning, further supporting that substantial repertoire restructuring occurs between Round 1 and Round 2.

Together, these findings demonstrate that PS-ASO selection induces a structured redistribution of clonal abundance, characterized not only by enrichment of individual sequences but also by a measurable increase in clonal dominance and reduced repertoire evenness.

### 3.5 Convergent physicochemical shifts among enriched CDR3-VH clonotypes

**Figure 5:**
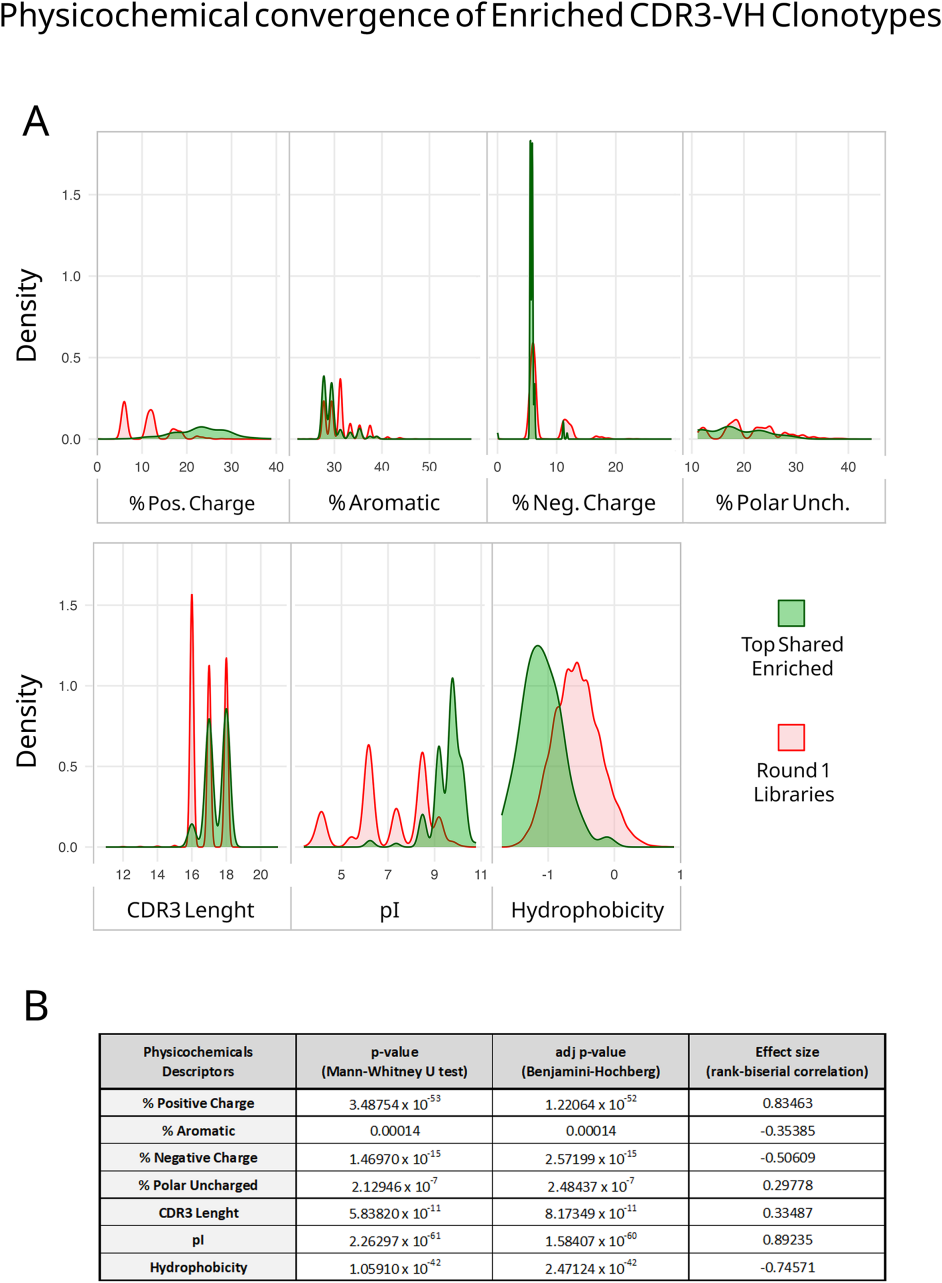
Global physicochemical convergence of enriched CDR3-VH clonotypes following PS-ASO selection. **(A)** Density distributions of physicochemical descriptors comparing the Round 1 reference repertoire (R1 libraries; red) with shared enriched CDR3-VH clonotypes (E_shared ≥ 4; N = 113; green). The enriched set was defined as clonotypes reproducibly enriched across the two independent selection strategies. Each unique clonotype was analyzed once, independently of its abundance, to evaluate intrinsic sequence-derived properties rather than expansion magnitude. Descriptors include the percentage of positively charged residues (K, R, H), negatively charged residues (D, E), aromatic residues (W, Y, F), polar uncharged residues (S, T, N, Q, C), FR3-CDR3-FR4 length, theoretical isoelectric point (pI), and mean hydrophobicity calculated using the Kyte–Doolittle scale. Shifts in distribution profiles indicate selection-associated remodeling of residues composition, loop length, pI, and hydrophilicity among enriched clonotypes. **(B)** Statistical comparison of physicochemical descriptors between enriched clonotypes and the R1 reference repertoire. *p*-values were calculated using the Mann–Whitney U test and adjusted for multiple comparisons using the Benjamini–Hochberg false discovery rate procedure (FDR). Rank-biserial correlation was reported as effect size ranges from -1 to 1, where 0 indicates no difference, and 1 or -1 indicates maximum differences between the experimental groups. Together, these analyses support convergence at the level of physicochemical properties rather than strict primary sequence identity.

To determine whether selective pressure driven by PS-ASOs resulted in recurrent sequence properties in addition to clonal dominance, we performed a physicochemical analysis of the enriched CDR3-VH clonotypes (**Figure 5**). The analysis was conducted on an unweighted basis, whereby each unique clonotype contributed once regardless of its abundance, allowing evaluation of intrinsic sequence properties independently of expansion magnitude.

The enriched population was defined as the set of shared clonotypes meeting the predefined threshold (E_shared ≥ 4; N = 113) across both independent selection strategies. The complete list of the 113 shared enriched CDR3-VH clonotypes, together with enrichment scores and physicochemical descriptors, is provided in **Supplementary Table 5**. To facilitate biological interpretation of this enriched set, **Supplementary Table 6** reports representative high-ranking clonotypes selected according to shared enrichment and distinctive physicochemical profiles. This table integrates E_shared values, fold enrichment and CPM levels in S1 and S2, highlighting clonotypes that were reproducibly enriched across both selection strategies and, in several cases, displayed particularly marked expansion in S2. For comparison, physicochemical properties were also computed for the reference repertoire corresponding to the R1 clonotypes prior to R2 selective enrichment.

For each shared clonotype sequence, quantitative descriptors were calculated, including the proportion of positively charged residues (K, R, H), negatively charged residues (D, E), aromatic residues (W, Y, F), and polar uncharged residues (S, T, N, Q, C), together with FR3-CDR3-FR4 length, theoretical isoelectric point (pI), and mean hydrophobicity computed according to the Kyte–Doolittle scale. Formal statistical comparisons between enriched and reference repertoires were performed for all descriptors using the Mann–Whitney U test with Benjamini–Hochberg FDR correction, and rank-biserial correlation was reported as effect size (**Figure 5B**).

These analyses identified significant shifts across the full descriptor panel, with particularly large effect sizes for positive charge, theoretical pI and hydrophobicity, supporting non-random physicochemical remodeling of the enriched CDR3-VH repertoire. Density distributions revealed consistent shifts between the reference R1 repertoires and the enriched clonotype population. Enriched clonotypes displayed a more defined charge composition, a clear CDR3 length distribution with preferential representation of 17–18 amino-acid CDR3 VH region and a shift toward more hydrophilic profiles. Importantly, these differences were reproducible across independent selection strategies, supporting the robustness of the observed enrichment patterns. It is important to note that the engineered CDR3-VH into the phage display library can be four, five, or six amino-acids long, plus 12 amino-acids retained from the invariable sequences FR3 and FR4, used along the bioinformatics pipeline. Accordingly, the observed length distribution reflects the full extracted CDR3-VH region analyzed in this study. Consistent with this trend, the enriched repertoire also exhibited a displacement toward higher theoretical pI values and reduced hydrophobicity, indicating an overall enrichment in polar and weakly basic sequence configurations. Despite these physicochemical shifts, sequence-level analysis revealed substantial heterogeneity among enriched clonotypes. Pairwise sequence similarity analysis showed that enriched clonotypes did not collapse into a single dominant sequence family (**Supplementary Figure 5**), supporting convergence at the level of physicochemical properties rather than strict primary sequence identity. Representative high-E_shared clonotypes showed that different residue combinations can produce comparable global physicochemical profiles, consistent with multiple sequence solutions compatible with PS-ASO recognition.

Together with the enrichment landscape (**Figure 3**) and rank–abundance analysis (**Figure 4**), these results indicate that selection against PS-ASOs induces both quantitative repertoire focusing and qualitative physicochemical convergence within the CDR3-VH repertoire. Importantly, this convergence emerges already at early stages of selection (R1–R2 transition), supporting the relevance of early repertoire remodeling in shaping candidate PS-ASO-reactive clonotypes. To further examine whether the global physicochemical shifts observed in enriched CDR3-VH clonotypes were associated with positional organization, we analyzed the variable CDR3-VH core after excluding invariant flanking residues. Because the randomized insert can contain 4, 5, or 6 amino-acids, sequences were grouped by core length before positional analysis. This analysis revealed non-random residue-class distributions and recurrent preferences for positively charged and aromatic residues within defined core positions (**Supplementary Figure 4**). Sequence-logo analysis confirmed that enriched clonotypes did not converge toward a single dominant motif, but rather displayed recurrent residue-class preferences compatible with physicochemical convergence. To quantitatively assess primary-sequence relatedness, pairwise Levenshtein distances were calculated among enriched CDR3-VH clonotypes. The resulting distance matrix showed broad sequence heterogeneity, supporting the conclusion that enriched clonotypes remain diverse at the primary sequence level despite converging in physicochemical descriptor space (**Supplementary Figure 5**).

A parallel analysis of enriched CDR3-VL clonotypes also revealed significant physicochemical remodeling (**Supplementary Figure 6**; **Supplementary Table 7**). Effect-size analysis indicated moderate shifts in positive charge, theoretical pI, polar uncharged residues, and hydrophobicity, suggesting that CDR3-VL is not neutral during selection and may contribute to the binding-competent physicochemical context of selected scFv fragments. However, the CDR3-VL profile did not reproduce the full convergence pattern observed for CDR3-VH, particularly with respect to aromatic enrichment. Thus, these data support a model in which CDR3-VL participates in physicochemical tuning of selected binders, whereas the most distinctive and mechanistically interpretable convergence signature remains primarily associated with CDR3-VH.

### 3.6 Functional validation supports an association between NGS-derived CDR3-VH features and preferential recognition of PS-modified oligonucleotides

**Figure 6:**
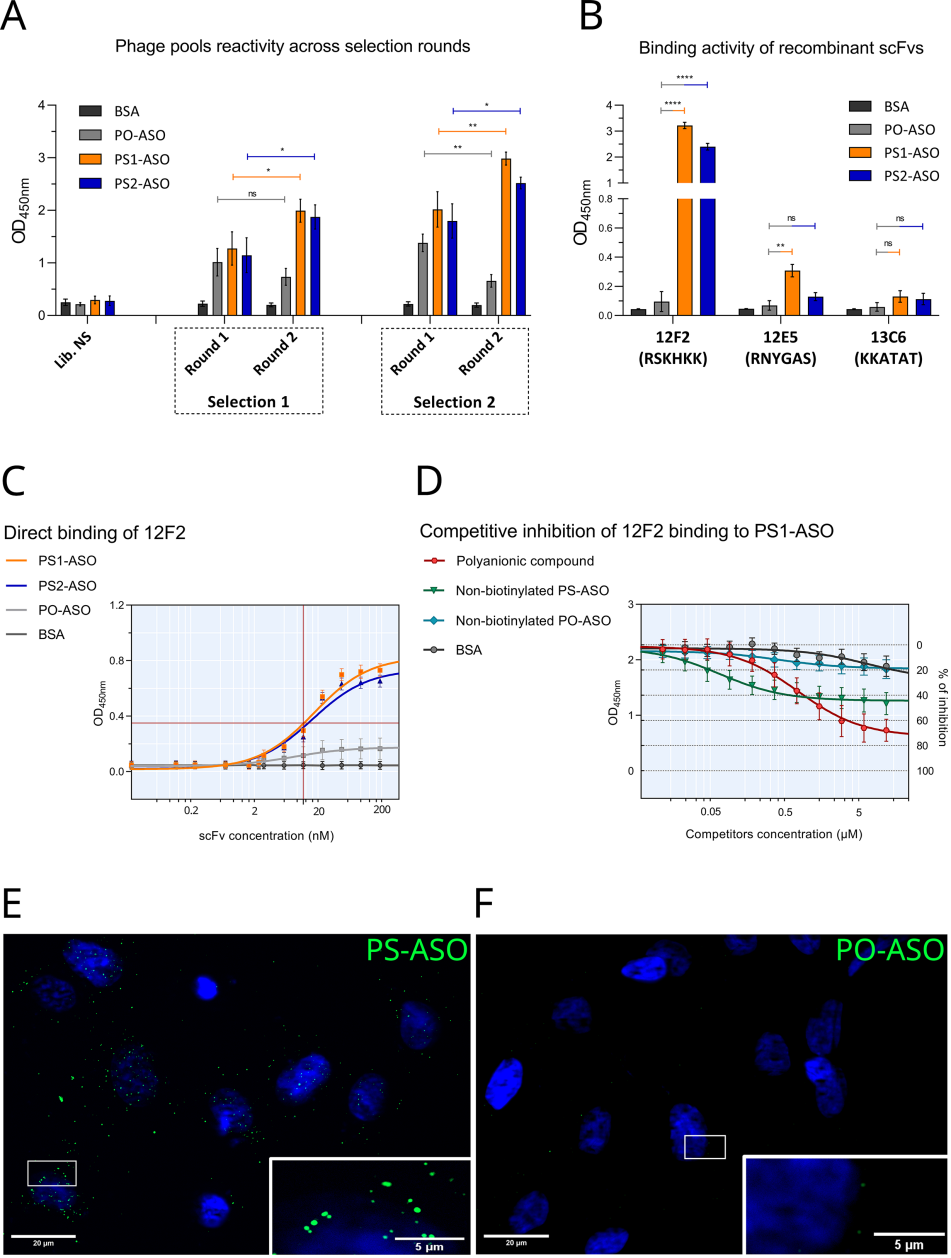
Functional validation of PS-ASO-reactive scFv clones derived from early selection rounds. **(A)** Phage ELISA analysis of selection outputs from Selection 1 and Selection 2 across Round 1 and Round 2. Reactivity was tested against BSA, PO-ASO, PS1-ASO, and PS2-ASO. Increased binding to PS-ASO targets from R1 to R2 supports functional enrichment during early biopanning and is consistent with the repertoire focusing detected by NGS. **(B)** Binding activity of representative recombinant scFv clones isolated from the Selection 2-Round 2 enriched repertoire. Clones were selected according to their degree of consistency with the NGS-derived CDR3-VH physicochemical signature. The CDR3-VH core amino-acidic sequence of clones 12F2, 12E5, and 13C6 is indicated in parentheses in the titles of the columns. Reactivity was tested against BSA, PO-ASO, PS1-ASO, and PS2-ASO. Clone 12F2 showed preferential binding to both PS-ASO targets, with minimal reactivity toward PO-ASO and BSA controls, whereas 12E5 and 13C6 displayed weaker or negligible PS-ASO-associated reactivity. **(C)** Dose-dependent binding of recombinant scFv 12F2 measured by direct ELISA. Binding curves were generated using increasing concentrations of scFv 12F2 against PS1-ASO, PS2-ASO, PO-ASO, and BSA. Curve interpolation from the dose–response plots identified an apparent ELISA-derived EC_50_ of approximately 7 nM for PS1-ASO binding, corresponding to half-maximal OD values. **(D)** Competitive inhibition of captured scFv 12F2 binding to PS1-ASO. Binding was evaluated in the presence of increasing concentrations of polyanionic compound, free non-biotinylated PS-ASO, free non-biotinylated PO-ASO, or BSA. Reduced binding in the presence of free PS-ASO and polyanionic compound supports the contribution of charged backbone-associated interactions to 12F2 recognition. **(E–F)** Representative cell-based detection assay following transfection with PS-ASO or PO-ASO. A detectable intracellular dotted signal was observed in PS-ASO-treated cells **(E)**, whereas PO-ASO-treated cells showed absent or nearly absent signal **(F)**. Nuclei are counterstained with the blue dye 4′,6-diamidino-2-phenylindole (DAPI). Scale bars: 20 µm in the main images and 5 µm in the magnified inserts. Data in ELISA panels are shown as mean ± SD of replicate measurements. Differences between groups were analyzed by unpaired (independent) two-tailed *t*-test, *p* ≥ 0.05 (ns) *p* < 0.05 (*), *p* < 0.01 (**), *p* < 0.001 (***), and *p* < 0.0001 (****).

To determine whether the repertoire changes detected by NGS were associated with functional binding activity, phage ELISA assays were performed using selection phage pools from both independent strategies and consecutive biopanning rounds. Reactivity toward PS-ASO targets increased from R1 to R2, indicating that the early repertoire focusing observed by sequencing was accompanied by increased functional binding activity (**Figure 6A**). Notably, Selection 2 showed higher reactivity than Selection 1, consistent with efficient enrichment under conditions involving sequence-independent PS-ASO targets.

Representative scFv clones were then isolated from Round 2 and selected according to their degree of consistency with the NGS-derived CDR3-VH physicochemical signature, as inferred from residue-class distribution and sequence-logo analysis of the enriched CDR3-VH core (**Supplementary Figure 4**). In particular, clone 12F2 (RSKHKK) showed the strongest agreement with the enriched profile, displaying multiple positively charged residues, mainly arginine and lysine, in positions compatible with the amino-acid distribution observed in the sequence logo, especially within the central region of the CDR3-VH. In contrast, clones 12E5 (RNYGAS) and 13C6 (KKATAT) showed more limited correspondence with the dominant logo features: 12E5 retained only a restricted positively charged component, whereas 13C6 contained N-terminal lysine residues but lacked the central enrichment of basic residues observed in the selected repertoire. Thus, these clones were used as representative variants with different degrees of conformity to the enriched CDR3-VH physicochemical profile rather than as exact positional matches to a fixed consensus sequence. The full-length sequences of the selected clones, obtained by Sanger sequencing, are reported in **Supplementary Table 8**.

Recombinant scFv fragments were tested by ELISA against PS1-ASO, PS2-ASO, PO-ASO and BSA. Among these clones, 12F2 showed preferential binding to both PS-ASO targets, with minimal reactivity toward PO-ASO and BSA controls (**Figure 6B**). These data support the interpretation that 12F2 is consistent with preferential recognition of shared PS-associated features rather than the nucleotide sequence of a single ASO target. Dose–response ELISA confirmed measurable binding of 12F2 to PS-ASO, with an ELISA-derived apparent EC_50_ in the low nanomolar range under these assay conditions (**Figure 6C**).

Competitive ELISA further showed that 12F2 binding was reduced by soluble competitors in a concentration-dependent manner. Inhibition was detectable at low competitor concentrations, starting from 0.05 µM for free non-biotinylated PS-ASO, and became more pronounced at higher concentrations, reaching approximately 70–75% with the polyanionic competitor and 50–60% with free PS-ASO (**Figure 6D**). Inhibition, on the other hand, is only weakly and detected at high concentrations for free PO-ASO and BSA, used as negative controls, suggesting a non-specific effect on binding. These findings are consistent with preferential recognition of PS-associated molecular features, while leaving open the contribution of additional structural determinants.

Finally, in a cell-based assay, cells transfected with PS-ASO showed a detectable, albeit moderate, intracellular signal, whereas PO-ASO-treated cells displayed absent or nearly absent signal (**Figures 6E, F**). Together, these data provide functional support for the NGS-derived observation that enrichment of specific CDR3-VH physicochemical features is associated with preferential recognition of PS-modified oligonucleotides.

## 4 Discussion

The present study provides quantitative evidence that selection against phosphorothioate-modified antisense oligonucleotides (PS-ASOs) is associated with reproducible antibody repertoire contraction and the emergence of clonal dominance across independent selection strategies. Repertoire diversity, enrichment profiles, and rank–abundance analyses consistently showed that repertoire focusing was already evident between Round 1 and Round 2. Thus, early stages of biopanning were sufficient to capture key features of selection-driven repertoire remodeling, although additional rounds and broader clone-level validation would be required to fully define high-affinity lead binders.

A key observation emerging from this study is that enriched clonotypes do not converge predominantly at the level of primary sequence. Instead, convergence occurs at the level of physicochemical properties within the CDR3-VH region, including an increased prevalence of positively charged residues, recurrent aromatic residue-class patterns, and a specific CDR3 length. This observation indicates that multiple distinct amino-acid sequences can converge toward similar physicochemical solutions compatible with interaction with phosphorothioate-modified oligonucleotides. This interpretation is further supported by the observation that selected clones displaying physicochemical features consistent with the enriched repertoire exhibit selective binding to PS-ASOs, while showing reduced or absent reactivity toward control targets. Together, these findings support the idea that the PS-backbone-associated features may contribute to selective pressure on the repertoire, favoring sequence-different clonotypes that share compatible physicochemical profiles.

The enrichment of overlapping clonotypes across two independent selection strategies, including one using PS-ASO targets with different nucleotide sequences but a shared phosphorothioate backbone, supports a major contribution of PS-backbone-associated molecular features rather than base sequence alone. However, a partial contribution of nucleotide sequence context cannot be excluded and will require further investigation. This interpretation is consistent with the known propensity of PS-modified oligonucleotides to engage protein surfaces through sequence-independent, property-driven interactions, suggesting that the convergence observed here reflects a broader principle of recognition of chemically modified polyanionic nucleic acids (3). Similar concepts regarding convergence of physicochemical configurations have been proposed in large-scale analyses of antibody repertoires, in which different sequences can converge toward compatible binding structures under selective pressure from common antigens (29, 30).

In this context, focusing on the heavy-chain complementarity-determining region 3 (CDR3-VH) is justified by its central role in binding specificity of semi-synthetic antibody libraries, particularly in the library used in this study, where most of the diversity is encoded within this region. The parallel analysis of CDR3-VL also revealed significant physicochemical remodeling, indicating that the light-chain CDR3 region is not neutral during selection and may contribute complementary features to PS-ASO recognition. Nevertheless, the most evident and mechanistically interpretable convergence signature remained associated with CDR3-VH in this library design.

Previous structural and structure–function studies of anti-DNA antibodies have shown that nucleic acid recognition frequently involves positively charged residues within CDR regions, particularly arginine and lysine, which contribute to interactions with the phosphate backbone, while aromatic residues can stabilize binding through base-stacking contacts. In particular, crystal structures of anti-ssDNA Fab complexes have shown that thymine bases can be accommodated between aromatic side chains, highlighting how sequence-diverse antibodies may exploit recurrent physicochemical features to engage nucleic acid targets (6, 7). The patterns observed here are compatible with these general principles, although PS-ASOs differ chemically and structurally from native DNA. Consistent with this interpretation, representative scFv 12F2 clone, recombinantly produced and tested by ELISA, exhibited preferential binding to both PS1-ASO and PS2-ASO, and minimal reactivity toward PO-ASO. From a broader biological perspective, these findings support the concept that antibody recognition of chemically modified oligonucleotides may be governed by general physicochemical constraints rather than strict primary sequence identity.

Functional validation was intentionally focused on representative selection outputs rather than exhaustive clone-level characterization. Nevertheless, the assays performed here support the functional relevance of the enriched repertoire, showing preferential recognition of PS-modified oligonucleotides by selected clones with CDR3-VH features consistent with the NGS-derived signature. This conclusion is supported by multiple controls, including bead pre-clearing, PO-ASO and BSA controls, competitive inhibition by free non-biotinylated PS-ASO and polyanionic competitor, and cell-based discrimination between PS-ASO and PO-ASO. Importantly, inhibition by the polyanionic DNA competitor indicates that electrostatic interactions contribute to binding, but this effect does not by itself account for the observed selectivity. The preferential binding to PS1-ASO and PS2-ASO, together with reduced reactivity toward PO-ASO and inhibition by free PS-ASO, supports recognition of PS-associated molecular features rather than nonspecific binding to nucleic acids in general. Further biochemical and structural analyses will help refine the binding mechanisms.

From an applied perspective, the identification of recurrent physicochemical signatures provides a conceptual framework for the rational design and prioritization of binding molecules targeting modified nucleic acids. Rather than focusing exclusively on individual sequences, future selection strategies could be guided by predefined physicochemical criteria, including charge distribution, aromatic content, hydrophilicity, and structural features of the binding loop. Consistent with previous observations on modified oligonucleotides, the recurrent enrichment of arginine and lysine residues suggests that positively charged side chains may contribute to recognition through favorable interactions with the modified backbone (31–33). The apparent convergence toward these interaction motifs across distinct binders raises the possibility that similar molecular principles could be used for the rational engineering of alternative recognition scaffolds beyond scFvs, with potential applications in both diagnostics and therapeutics. Such an approach may enable more efficient identification of candidate functional binders and may extend beyond antibody fragments to other classes of binding molecules (34).

Overall, these results indicate that selection against PS-ASOs promotes convergence toward restricted physicochemical configurations within the antibody repertoire, highlighting the role of target-imposed constraints in shaping binding architectures. This study therefore provides a conceptual and experimental framework for investigating and prioritizing antibody recognition of chemically modified nucleic acids.

## Data Availability

Raw sequencing reads are publicly available ( SRA BioProject: PRJNA1462733 – SRA link: https://trace.ncbi.nlm.nih.gov/Traces?study=SRP697998). Custom scripts used in this study—including the data science packages and tools—are available in the associated public GitHub data repository (https://github.com/FrancescoCoppolino/Physicochemical-convergence-in-antibody-CDR3-VH-repertoires-recognizing-PS-modified-ASO-backbone). This repository also includes processed clonotype abundance tables for each selection round. Selection 1, Rounds 1 and 2, correspond to A3I and A3II, respectively, and Selection 2, Rounds 1 and 2, correspond to A4I and A4II, respectively. Additional data will be made available upon request.
