# Supplementary material for: Physicochemical convergence in antibody CDR3-VH repertoires recognizing phosphorothioate-modified oligonucleotides backbone
Source: Front Immunol. 2026 May 29;17:1843118. doi: 10.3389/fimmu.2026.1843118 (PMC13259659; doi:10.3389/fimmu.2026.1843118)
Supplement: Supplementary file 1 [file DataSheet1.pdf]

# Supplementary Material

## 1 Supplementary Tables

**Supplementary Table S1. Sequencing output and processing metrics for VH and VL amplicon libraries.** Summary of sequencing output and processing metrics for each VH and VL amplicon library. The table reports raw sequencing reads, trimmed reads, successfully merged reads, nucleotide sequences after cut, translated amino-acidic sequences and final unique clonotype counts. Metrics are shown separately for each selection strategy, biopanning round, chain type, and read direction where applicable.

**Supplementary Table S2**. **Sensitivity analysis of cumulative read-count thresholds for CDR3-VH enrichment analysis.** Sensitivity analysis evaluating the effect of cumulative read-count filtering thresholds on clonotype retention and enrichment detection in CDR3-VH repertoires. Thresholds tested included R1 + R2 ≥ 2, ≥ 5, ≥ 10, and ≥ 20. For each independent selection strategy, the table reports the total number of CDR3-VH amino-acidic sequences, the number of clonotypes retained after filtering, and the number of enriched clonotypes identified using the predefined enrichment criterion. This analysis supports the choice of R1 + R2 ≥ 5 as a conservative threshold that reduces low-count stochastic noise while preserving sufficient repertoire diversity for downstream enrichment and physicochemical analyses.

**Supplementary Table S3**. **Sensitivity analysis of cumulative read-count thresholds on the shared enriched CDR3-VH clonotype set.** Sensitivity analysis evaluating the effect of cumulative read-count filtering thresholds on the identification of shared enriched CDR3-VH clonotypes. Thresholds tested included R1 + R2 ≥ 2, ≥ 5, ≥ 10, and ≥ 20. The table reports the number of clonotypes retained in the shared enriched set using the predefined criterion E_shared ≥ 4. The stability of the enriched set across thresholds from R1 + R2 ≥ 2 to R1 + R2 ≥ 10 supports the robustness of the selected cutoff, whereas the reduction observed at R1 + R2 ≥ 20 reflects the expected loss of lower-abundance but reproducibly enriched clonotypes under a more stringent filtering condition.

**Supplementary Table S4. Sensitivity analysis of E_shared thresholds for physicochemical descriptor comparisons.** Sensitivity analysis evaluating the robustness of physicochemical descriptor comparisons across different E_shared thresholds used to define shared enriched CDR3-VH clonotypes. For each threshold, the table reports the number of retained CDR3-VH clonotypes

and the statistical comparison of sequence-derived descriptors between enriched clonotypes and the R1 reference repertoire. Descriptors include percentage of positively charged residues, aromatic residues, negatively charged residues, polar uncharged residues, CDR3 length, theoretical isoelectric point (pI), and hydrophobicity. P-values were calculated using the Mann–Whitney U test and corrected for multiple comparisons using the Benjamini–Hochberg false discovery rate procedure. Rank-biserial correlation is reported as effect size. The persistence of significant shifts across threshold conditions supports the robustness of the physicochemical convergence observed in the enriched repertoire.

**Supplementary Table S5. Complete list of shared enriched CDR3-VH clonotypes identified across independent PS-ASO selection strategies.** Complete list of the 113 shared enriched CDR3-VH clonotypes identified using the predefined E_shared threshold (E_shared ≥ 4). For each clonotype, the table reports the CDR3-VH amino-acid sequence, enrichment values across independent selection strategies, shared enrichment score, and corresponding physicochemical descriptors, including residue composition, CDR3 length, theoretical isoelectric point, and hydrophobicity.

**Supplementary Table S6. Top representative shared enriched CDR3-VH clonotypes and distinctive physicochemical features.** Top representative shared enriched CDR3-VH clonotypes selected from the complete enriched set according to shared enrichment score. For each clonotype, the table reports distinctive physicochemical features, fold enrichment and CPM levels in S1 and S2. The table highlights representative sequence configurations characterized by high E_shared values, defined CDR3 length, increased positive charge, aromatic residue content, elevated theoretical pI, and hydrophilic profile. This table provides representative examples of sequence-diverse clonotypes converging toward similar physicochemical properties.

**Supplementary Table S7**. **Complete list of enriched CDR3-VL clonotypes identified across PS-ASO selection strategies**. Complete list of the 207 shared enriched CDR3-VLclonotypes identified using the predefined E_shared threshold (E_shared ≥ 4). For each clonotype, the table reports the CDR3-VL amino-acid sequence, enrichment values across independent selection strategies, shared enrichment score, and corresponding physicochemical descriptors, including residue composition, theoretical isoelectric point, and hydrophobicity.

**Supplementary Table S8. Sanger-confirmed full-length sequences of scFv clones 12F2, 12E5 and 13C6.** Amino-acid sequence obtained by Sanger sequencing, from the first methionine of the scFvs to the stop codon downstream of the 6*His tag. The amino-acids in bold represent the two regions FR3-CDR3-FR4, corresponding to VH and VL, respectively. The underlined amino-acids correspond to CDR3-VH and CDR3-VL, respectively. The linker region between VH chain and VL chain is shown in italic.

## 2 Supplementary captions

**Supplementary Figure S1. Amplicon design and NGS library preparation strategy for CDR3-VH and CDR3-VL repertoire profiling. (A)** Schematic representation of the phagemid containing scFv used as source of template DNA. VH and VL chains are marked in orange and blue,

respectively. **(B)** Three-step PCR strategy used for targeted amplification and library preparation. CDR3 target region is marked in light-orange and light-blue for VH and VL respectively. In the first PCR, gene-specific primers annealing to FR3 and FR4 invariable sequences amplify the FR3–CDR3–FR4 segments of the VH (P1 and P2) and VL (P3 and P4) chains. In the second PCR, Primers-pools incorporating heterogeneity spacers (0 to 5 N degenerated bases, in grey) and Illumina consensus sequences (Rd1 and Rd2 SP in yellow and cyan, respectively) were introduced, individually for VH-derived amplicons using VH primer pool, and for VL-derived amplicons using VL primer pool. In the third PCR, Illumina adapters (p5 in red and p7 in green) and dual-index barcodes (barcode box) are added to enable multiplexed sequencing. **(C)** Final indexed amplicons were pooled and subjected to paired-end Illumina sequencing.

**Supplementary Figure S2. Sample-level Phred quality metrics for VH and VL amplicon libraries.** Mean Phred quality scores across both forward and reverse reads obtained with a paired-end sequencing on MiSeq platform. Simple bars represent Sequencing Reads 1, and striped bars represent Sequencing Read 2. Error bars indicate the standard deviation across read positions. Samples (S) 1 to 5 correspond to VH -derived libraries respectively from Selection 1-Round 1, Selection 1-Round 2, Selection 2-Round 1, Selection 2-Round 2 and LibNS. Samples 6 to 10 correspond to VL-derived libraries of the same phage pools mentioned above. Overall, all libraries showed mean Phred quality values compatible with high-quality sequencing, supporting the technical consistency of the datasets used for downstream CDR3 clonotype reconstruction.

**Supplementary Figure S3. Enrichment distribution of CDR3-VH and CDR3-VL clonotypes relative to the non-selected library.** Distribution of clonotype enrichment values relative to the non-selected scFv phage display library (LibNS) across Selection 1 and Selection 2. CDR3-VH and CDR3-VL repertoires are shown separately for each selection strategy. Red circles indicate Round 1 clonotypes, whereas green circles indicate Round 2 clonotypes. Enrichment values are expressed relative to LibNS and displayed on a $\log_{10}$ scale. The upward shift and broader distribution observed in Round 2, particularly in Selection 2, indicate progressive enrichment of selected clonotypes relative to the starting library. This analysis complements the R2/R1 enrichment landscape shown in Figure 3 by providing an additional reference to the unselected repertoire.

**Supplementary Figure S4. Positional organization and sequence-logo analysis of enriched CDR3-VH clonotypes.** Residue-class distribution and sequence-logo analysis of the variable CDR3-VH core among shared enriched clonotypes (N=113). Invariant flanking residues were excluded before analysis, and sequences were grouped according to randomized core length to avoid alignment artifacts introduced by variable insert size (6 amino-acids core N=54, 5 amino-acids core N=50, 4 amino-acids core N=9). **(A)** Relative abundance of amino-acid classes across CDR3-VH core positions. Bars indicate the percentage contribution of each residue class at each position within the corresponding core-length group. **(B)** Sequence-logo representation of enriched CDR3-VH core residues, showing position-specific residue preferences. The analysis highlights recurrent residue-class organization among enriched clonotypes, particularly involving positively charged and structurally informative residues, while confirming the absence of a single dominant primary sequence motif.

**Supplementary Figure S5. Pairwise Levenshtein distance analysis of shared enriched CDR3-VH clonotypes.** Heatmap and hierarchical clustering based on pairwise Levenshtein distances among the 113 shared enriched CDR3-VH clonotypes. Distances were calculated on CDR3-VH amino-acid sequences after exclusion of invariant flanking residues where applicable. The broad distribution of

distance values and the absence of a single compact sequence cluster indicate that enriched clonotypes remain heterogeneous at the primary sequence level. These data support the conclusion that PS-ASO selection promotes convergence mainly at the level of physicochemical properties rather than strict sequence identity.

**Supplementary Figure S6. Physicochemical profiling of enriched CDR3-VL clonotypes across PS-ASO selection strategies. (A)** Physicochemical descriptor analysis of enriched CDR3-VL clonotypes identified across the PS-ASO selection datasets. Descriptor distributions include residue-class composition, theoretical isoelectric point, hydrophobicity, and CDR3-VL-related sequence features where applicable. **(B)** Statistical comparison of physicochemical descriptors between enriched clonotypes and the R1 reference repertoire. *p*-values were calculated using the Mann–Whitney U test and adjusted for multiple comparisons using the Benjamini–Hochberg false discovery rate procedure (FDR). Rank-biserial correlation was reported as effect size ranges from -1 to 1, where 0 indicates no difference, and 1 or -1 indicates maximum differences between the experimental groups. Compared with the CDR3-VH repertoire, enriched CDR3-VL clonotypes showed detectable but overall less pronounced physicochemical shifts and effect size ranges, supporting a complementary contribution of the light chain to selected scFv binding profiles while indicating that the dominant convergence signature is mainly associated with CDR3-VH.
