# Supplementary figures and images for: Physicochemical convergence in antibody CDR3-VH repertoires recognizing phosphorothioate-modified oligonucleotides backbone
Source: Front Immunol. 2026 May 29;17:1843118. doi: 10.3389/fimmu.2026.1843118 (PMC13259659; doi:10.3389/fimmu.2026.1843118)

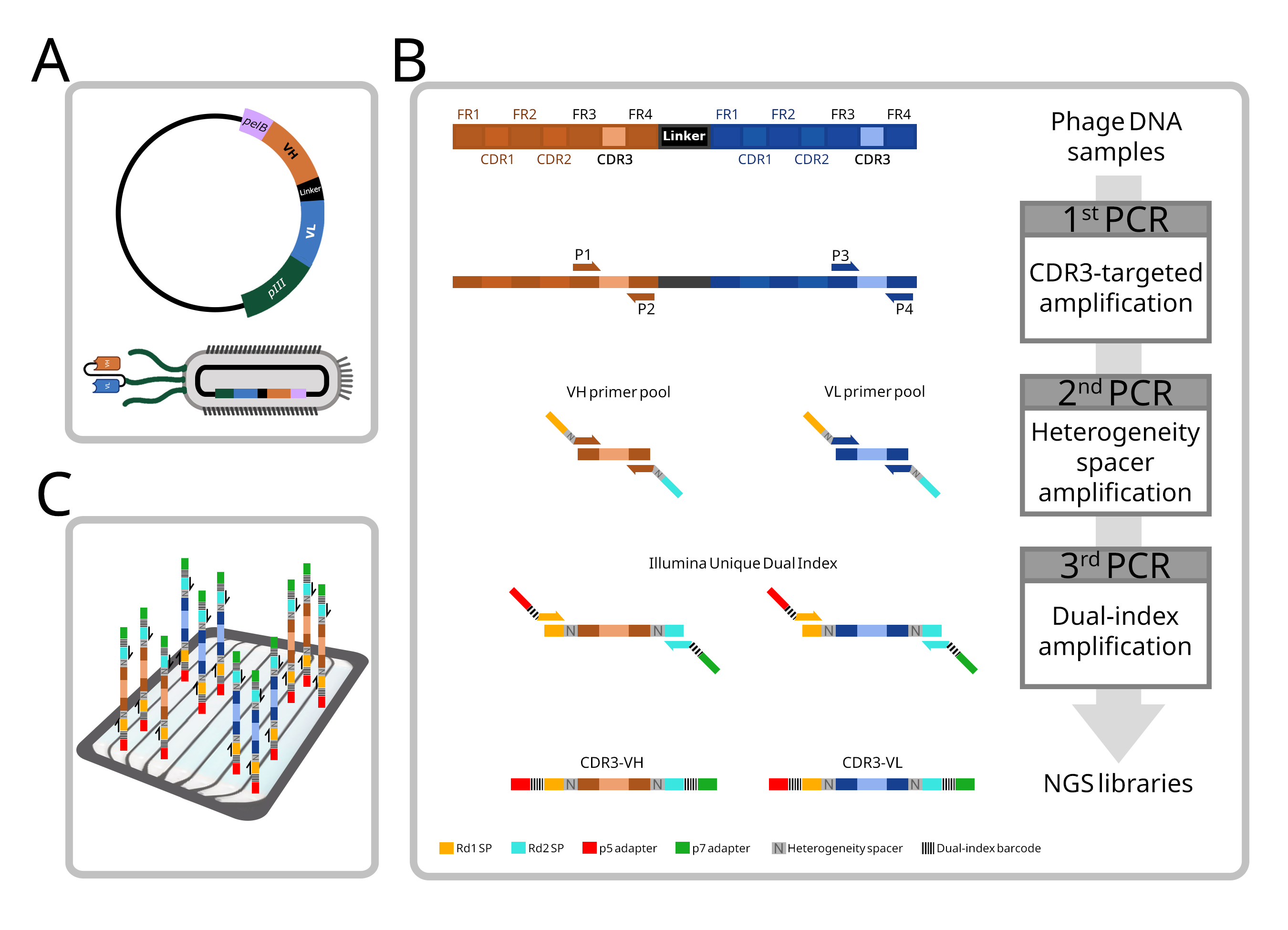

Supplement: Supplementary file 2 [file Image1.tif]

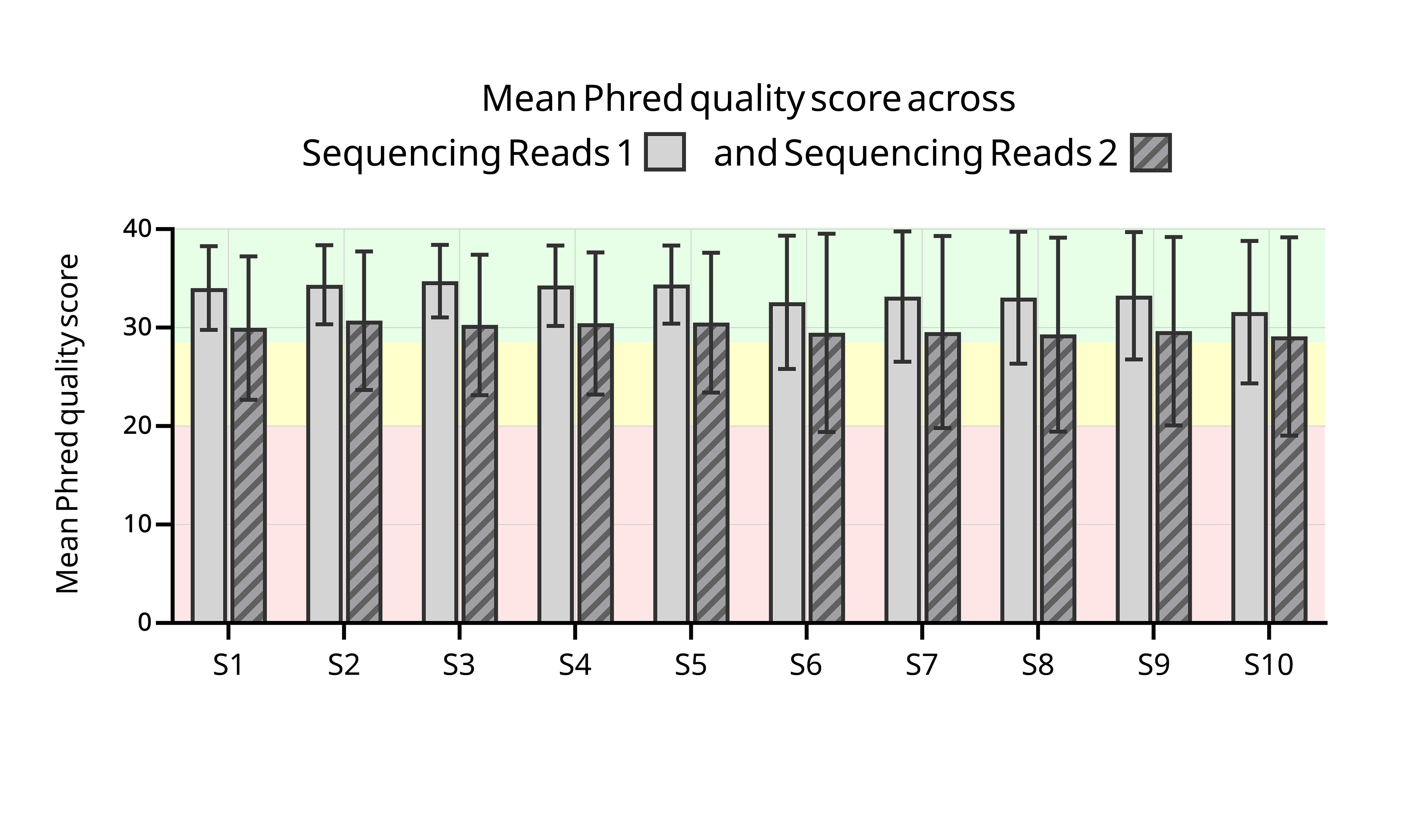

Supplement: Supplementary file 3 [file Image2.tif]

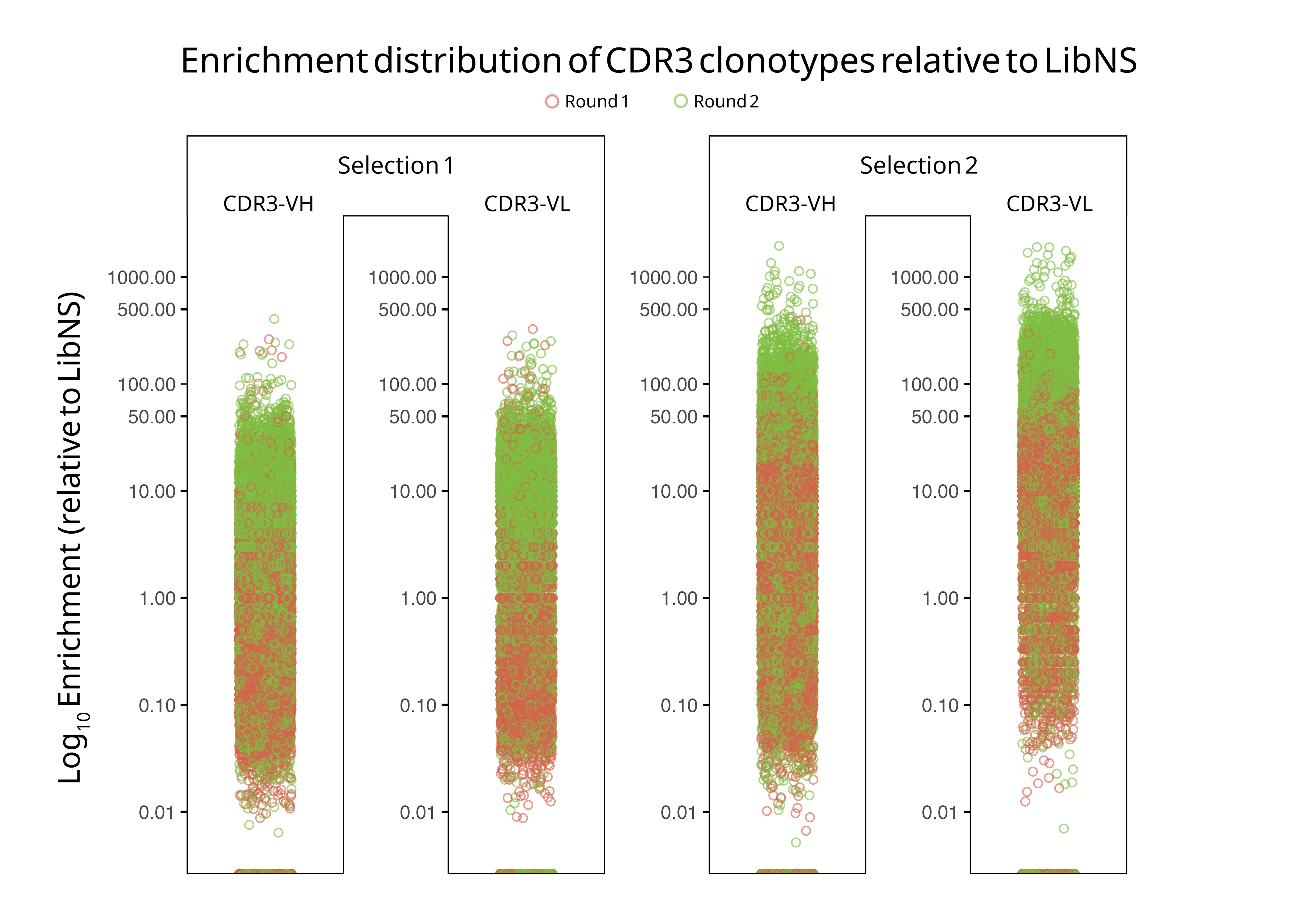

Supplement: Supplementary file 4 [file Image3.tif]

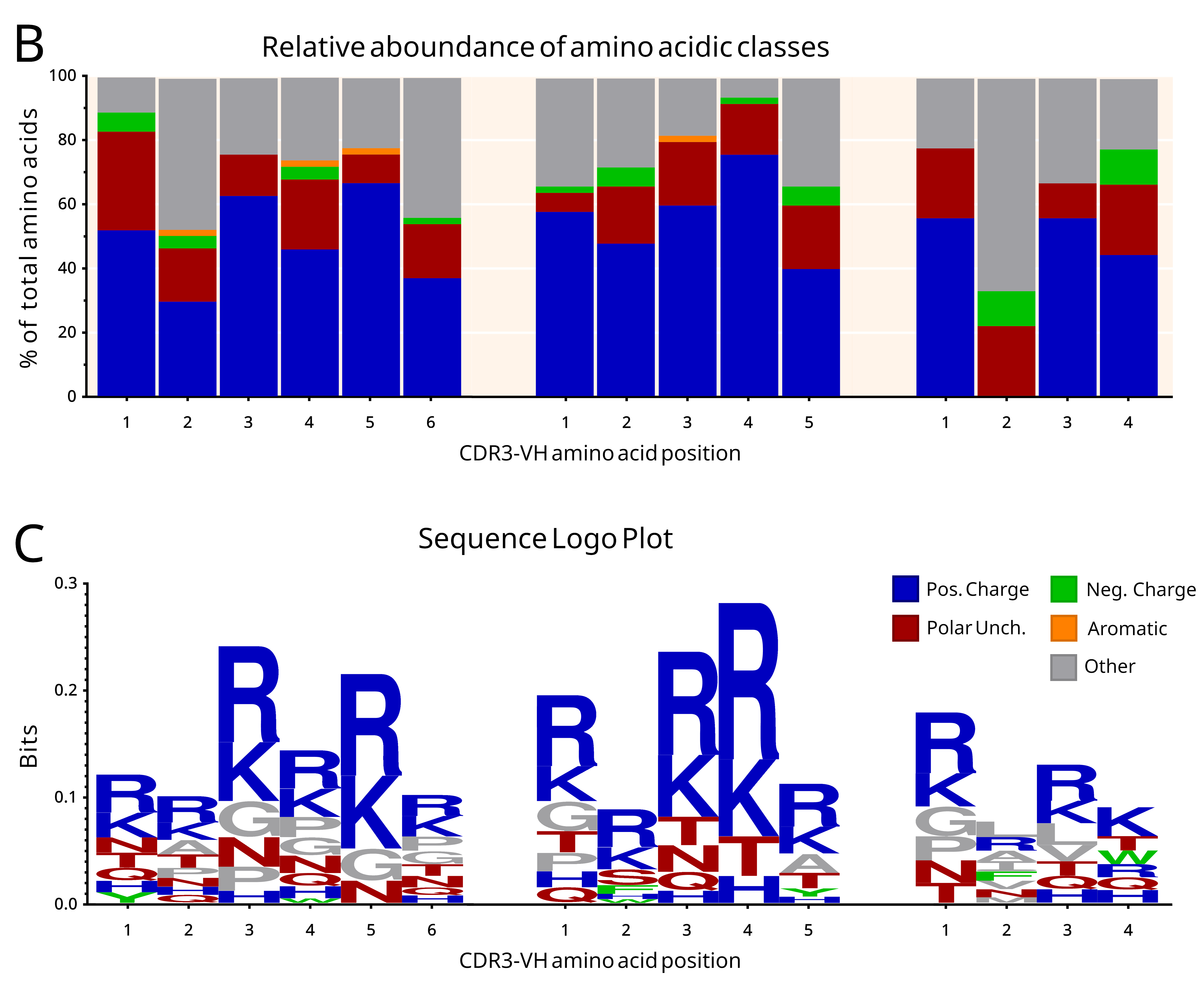

Supplement: Supplementary file 5 [file Image4.tif]

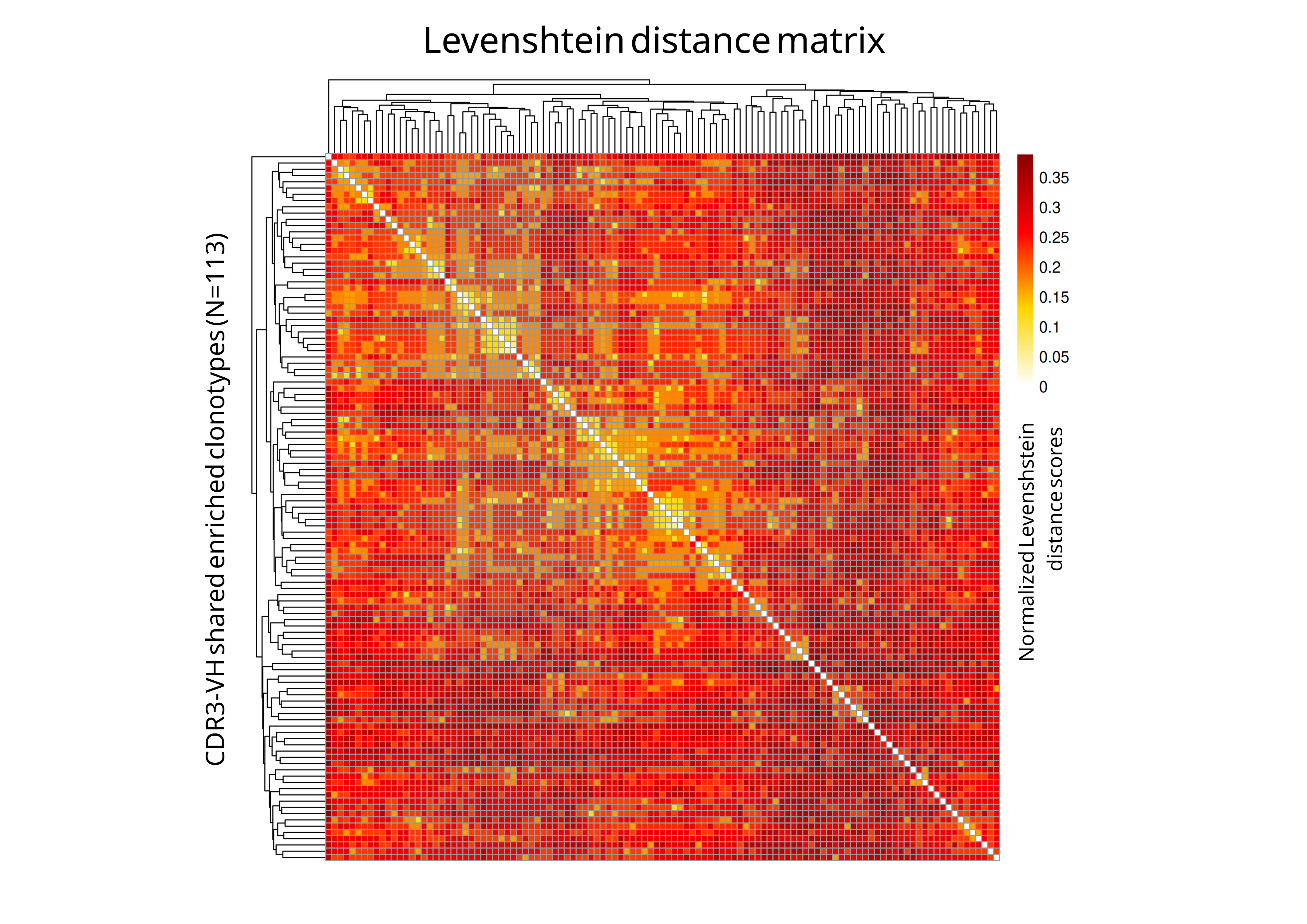

Supplement: Supplementary file 6 [file Image5.tif]

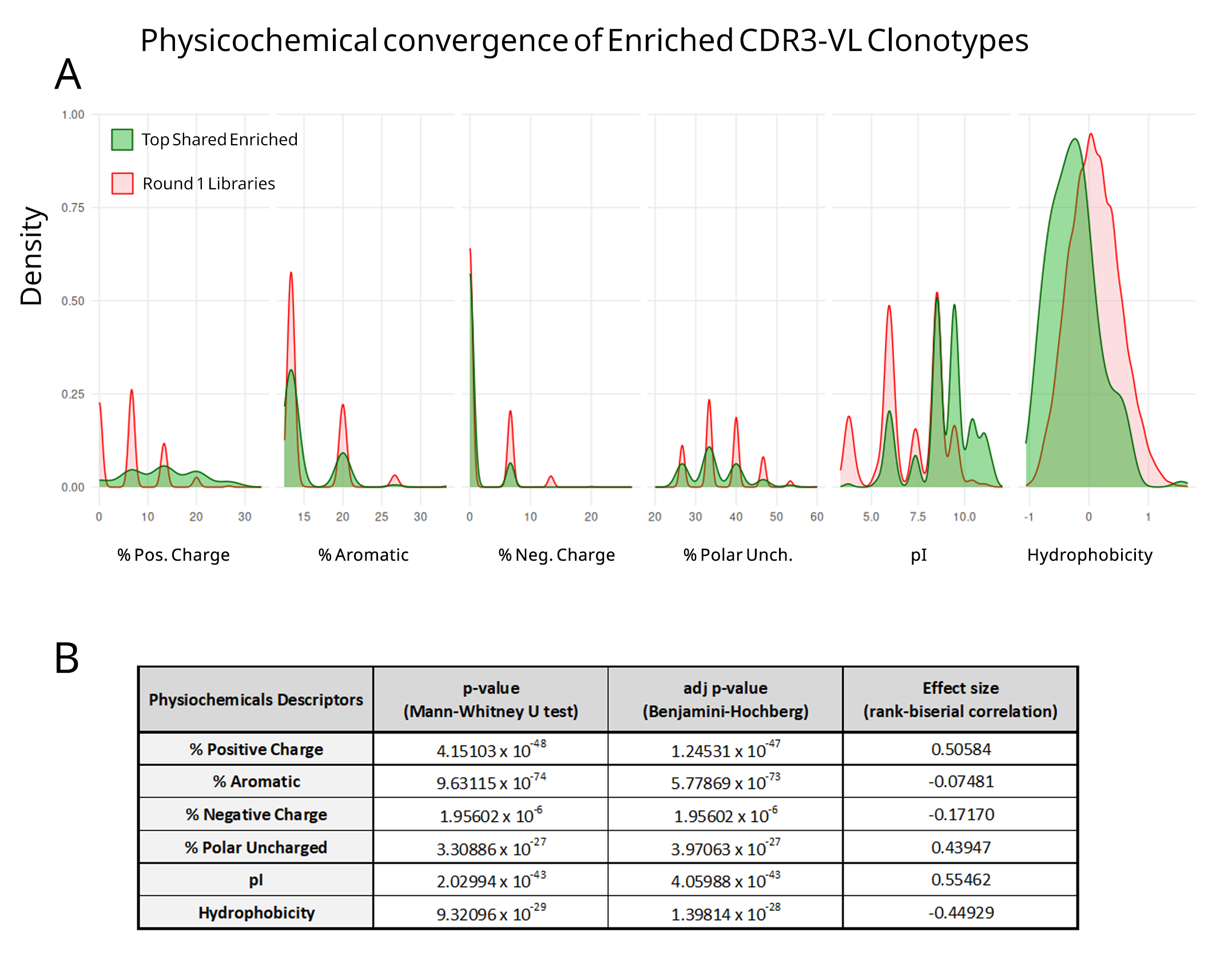

Supplement: Supplementary file 7 [file Image6.tif]
